# Supplementary material for: Integrating spin-dependent emission and dielectric switching in FeII catenated metal-organic frameworks
Source: Nat Commun. 2024 May 10;15:3961. doi: 10.1038/s41467-024-48425-8 (PMC11087595; doi:10.1038/s41467-024-48425-8)
Supplement: Supplementary file 2 — Supplementary Information [file 41467_2024_48425_MOESM2_ESM.pdf]

Supplementary Information

Integrating spin-dependent emission and dielectric switching in Fe^{II} catenated metal-organic frameworks

Xue-Ru Wu,[1] Shu-Qi Wu,*,[2] Zhi-Kun Liu,[3] Ming-Xing Chen,[4] Jun Tao,[3] Osamu Sato[2], and Hui-Zhong Kou*,[1]

[1] Department of Chemistry, Tsinghua University, Beijing 100084, P. R. China.

[2] Institute for Materials Chemistry and Engineering & IRCCS, Kyushu University, 744 Motooka, Nishi-ku, Fukuoka 819-0395, Japan.

[3] Key Laboratory of Cluster Science of Ministry of Education, School of Chemistry and Chemical Engineering, Beijing Institute of Technology, Liangxiang Campus, Beijing 102488, P. R. China.

[4] Analytical Instrumentation Center, College of Chemistry and Molecular Engineering, Peking University, Beijing 100871, P. R. China.

Correspondence to: wu.shuqi.152@m.kyushu-u.ac.jp; kouhz@mail.tsinghua.edu.cn

Supplementary Table 1 Crystal data and structure refinement for 1Ag.

T / K	100	170	250
Formula	$C_{33}H_{19}Ag_2Cl_9FeN_8O_4$	$C_{33}H_{19}Ag_2Cl_9FeN_8O_4$	$C_{33}H_{19}Ag_2Cl_9FeN_8O_4$
Formula weight	1182.20	1182.20	1182.20
Crystal system	tetragonal	tetragonal	tetragonal
Space group	$I4_1cd$	$I4_1cd$	$I4_1cd$
a / Å	20.75640(10)	20.97220(10)	21.05001(15)
b / Å	20.75640(10)	20.97220(10)	21.05001(15)
c / Å	36.4876(5)	36.6216(4)	36.8308(6)
α, β, γ / °	90	90	90
V / Å^3	15719.9(3)	16107.4(2)	16319.8(3)
Z	16	16	16
ρ_{calc} /g cm^{-3}	1.998	1.950	1.925
F (000)	9248.0	9248.0	9248.0
Radiation	Cu Kα	Cu Kα	Cu Kα
GOF on F^2	1.141	1.036	1.082
R_1 [I >=2$\sigma(I)$]^a	0.0568	0.0521	0.0449
wR_2 [all data]	0.1627	0.1497	0.1312
R_{int}	0.0493	0.0416	0.0209
CCDC	2249941	2249940	2249939

^{a} $R_1=\sum \| F_o|-|F_c \| / \sum|F_o|$. $wR_2=\left\{\sum\left[w\left(F_o^2-F_c^2\right)^2\right]/\sum\left[w\left(F_o^2\right)^2\right]\right\}^{1/2}$

Supplementary Table 2 Crystal data and structure refinement for 1Au and 2Ag.

	**1Au**		**2Ag**
T / K	100	220	173
Formula	$C_{32}H_{22}Au_2Cl_6FeN_8O_6$	$C_{32}H_{22}Au_2Cl_6FeN_8O_6$	$C_{33}H_{19}Ag_2Cl_9N_8O_4Zn$
Formula weight	1277.06	1277.06	1191.72
Crystal system	orthorhombic	orthorhombic	tetragonal
Space group	*Ccce*	*Ccce*	$I4_1cd$
a / Å	18.6620(3)	18.798(4)	20.96475(18)
b / Å	20.1728(4)	20.958(4)	20.96475(18)
c / Å	20.6673(4)	20.444(4)	36.6689(6)
α, β, γ / °	90	90	90
V / Å^3	7780.5(3)	8054(3)	16116.8(4)
Z	8	8	16
ρ_{calc} / g cm^{-3}	2.180	2.106	1.965
F (000)	4832	4832	9312
Radiation	Cu Kα	Cu Kα	Cu Kα
GOF on F^2	1.075	1.125	1.056
R_1 [I >=2σ(I)]	0.0858	0.0572	0.0575
wR_2 [all data]	0.2195	0.1897	0.1556
R_{int}	0.0279	0.0343	0.0320
CCDC	2249943	2249942	2269751

Supplementary Table 3 Selected bond lengths (Å) for 1Ag.

T / K	100	170	250
Fe1-N1	2.023(9)	2.086(7)	2.110(6)
Fe1-N2#1	2.013(9)	2.074(8)	2.106(6)
Fe1-N3	2.120(10)	2.185(8)	2.195(6)
Fe1-N4#2	2.103(10)	2.181(8)	2.204(6)
Fe1-N5	2.102(10)	2.202(8)	2.215(7)
Fe1-N6	2.132(10)	2.210(8)	2.242(7)
Ag1-C1	2.043(10)	2.027(9)	2.037(7)
Ag1-C2	2.041(11)	2.030(9)	2.049(7)
Ag2-C3	2.083(12)	2.083(9)	2.086(7)
Ag2-C4	2.089(13)	2.075(10)	2.083(8)

Symmetry codes: #1 -1/2+x, 1/2-y, +z; #2 1/2-x, 1/2+y, +z.

Supplementary Table 4 Selected bond lengths (Å) for 1Au at 100 K.

Fe1-N1	2.065(10)	Fe1-N1#1	2.065(10)
Fe1-N2	2.054(16)	Fe1-N3#2	2.038(13)
Fe1-N4	2.133(9)	Fe1-N4#1	2.133(9)
Au1-C1	1.983(13)	Au1-C1#3	1.983(13)
Au2-C2	2.004(16)	Au2-C3	1.99(2)

Symmetry codes: #1 3/2-x, -y, +z; #2 +x, -y, 1/2+z; #3 3/2-x, 1/2-y, 1-z.

Supplementary Table 5 Selected bond lengths (Å) for 1Au at 220 K.

Fe1-N1	2.093(12)	Fe1-N2#2	2.081(10)
Fe1-N3	2.224(7)	Fe1-N3#2	2.224(7)
Fe1-N4	2.223(6)	Fe1-N4#2	2.223(6)
Au1-C1	1.929(15)	Au1-C2	1.934(16)
Au2-C3	2.027(10)	Au2-C3#3	2.027(10)

Symmetry codes: #1 +x, 1-y, 1/2+z; #2 3/2-x, 1-y, +z; #3 3/2-x, 1/2-y, -z.

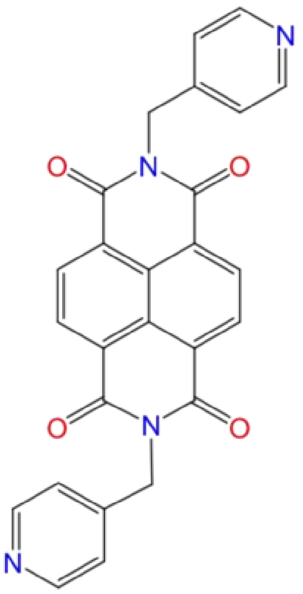

Supplementary Figure 1 Molecular structure of BPND ligand. BPND ligand is used in the synthesis of the title complexes.

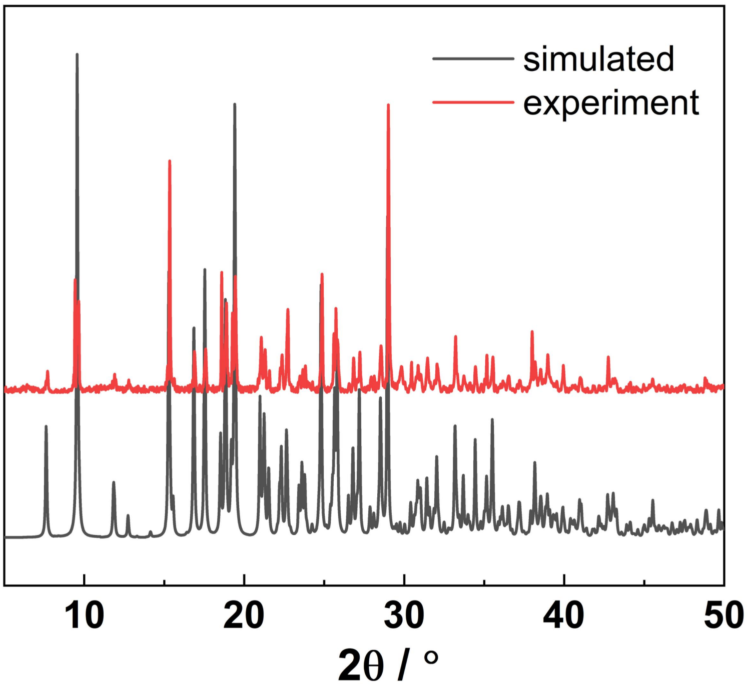

Supplementary Figure 2 Powder X-ray diffraction patterns for 1Ag. Comparison of experimental (room temperature) and simulated peaks indicates bulk phase purity of the polycrystalline sample.

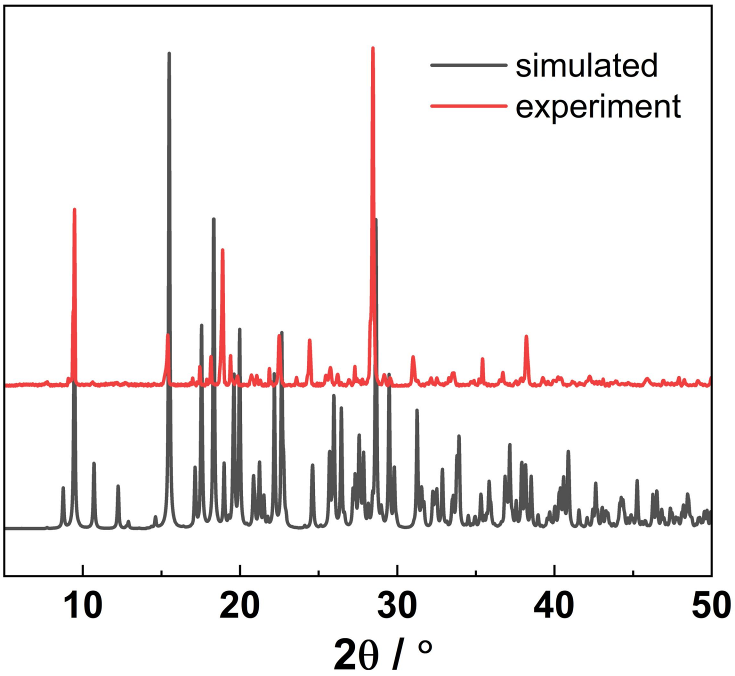

Supplementary Figure 3 Powder X-ray diffraction patterns for 1Au. Comparison of experimental (room temperature) and simulated peaks indicates bulk phase purity of the polycrystalline sample.

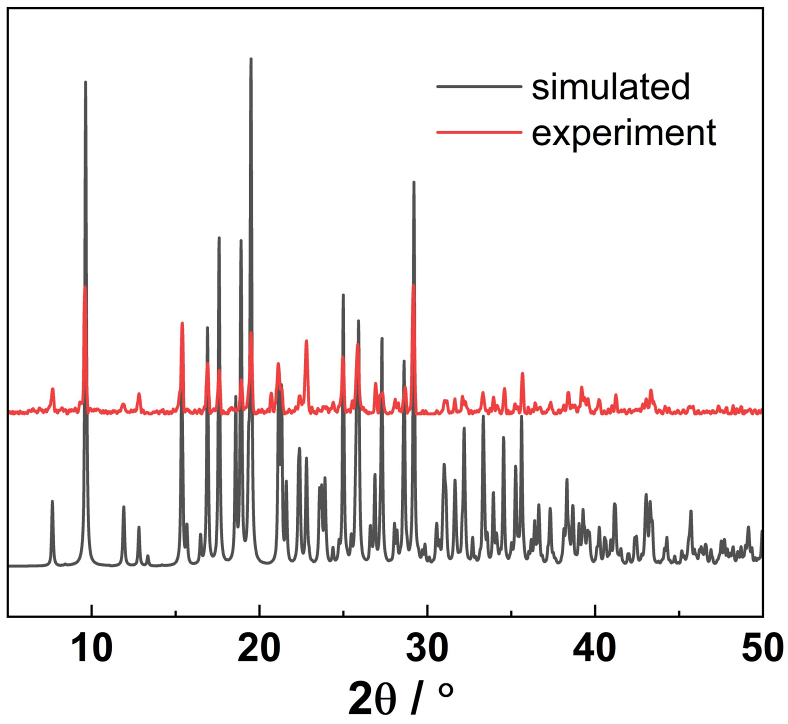

Supplementary Figure 4 Powder X-ray diffraction patterns for 2Ag. Comparison of experimental (room temperature) and simulated peaks indicates bulk phase purity of the polycrystalline sample.

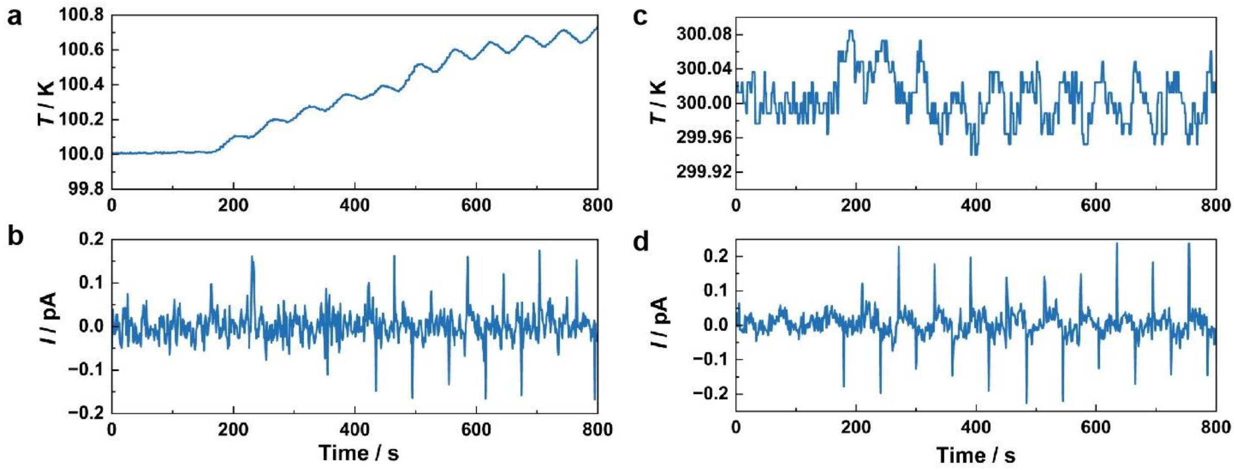

Supplementary Figure 5 Pyroelectric properties for 1Ag. Raw data of photo-pyroelectric measurements in the MPMS-XL chamber of **1Ag** at 100 K (a, b) and 300 K (c, d), respectively. (a and b panels show the temperature and pyroelectric current, respectively).

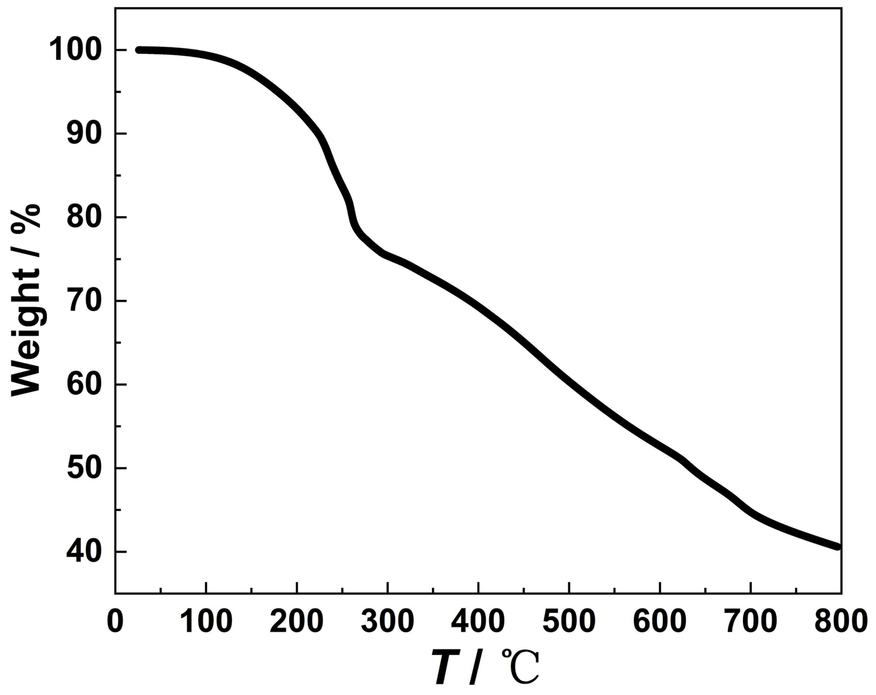

Supplementary Figure 6 Thermogravimetric analysis of 1Ag. The desolvation process at high temperature (> 270 ºC) is accompanied by the weight loss due to decomposition.

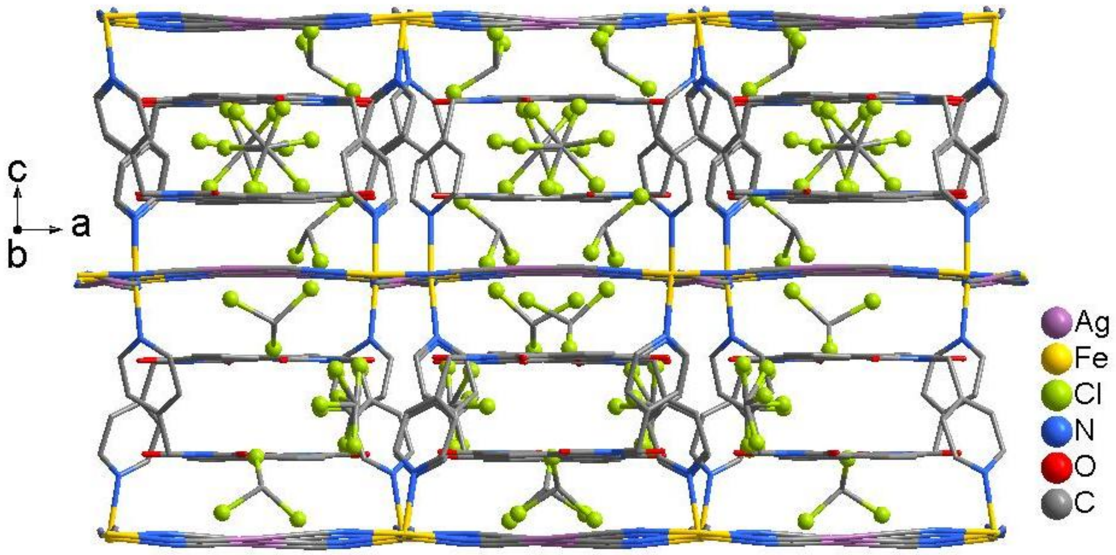

Supplementary Figure 7 Packing modes of 1Ag. Cell packing diagram revealing intermolecular π-π stacking along the *b*-axis direction for **1Ag**. Hydrogen atoms are omitted for clarity.

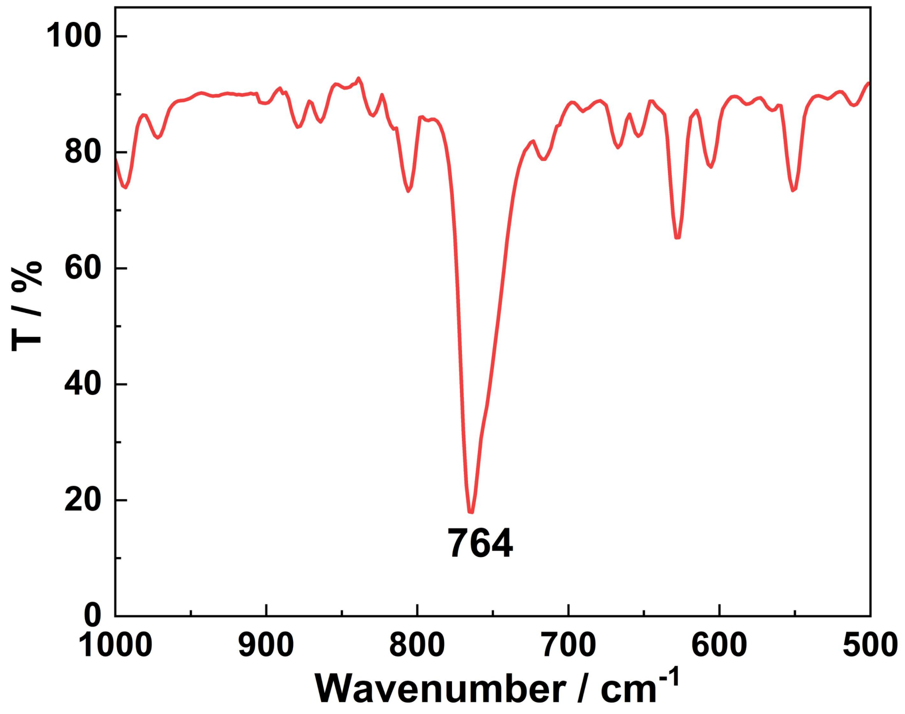

Supplementary Figure 8 IR spectrum of 1Ag. The presence of chloroform is verified by the strong peak at 764 cm^{-1} for C–Cl stretching vibration.

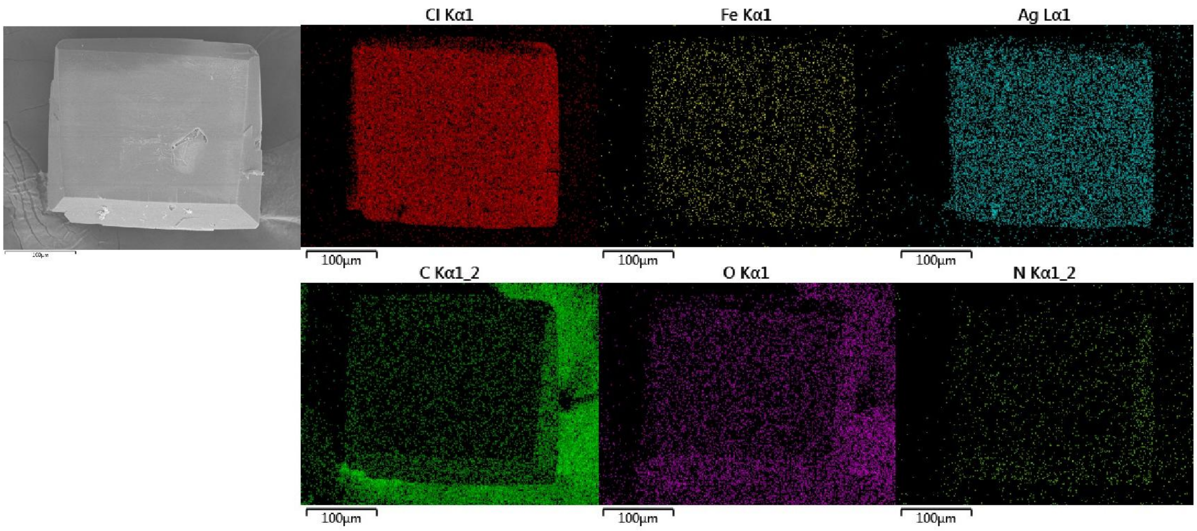

Supplementary Figure 9 SEM image and elemental mapping photographs of 1Ag (single crystal). The elemental mapping images show that Cl, Fe, Ag, C, O, and N are evenly distributed in the crystal, indicating the presence of solvent chloroform molecules.

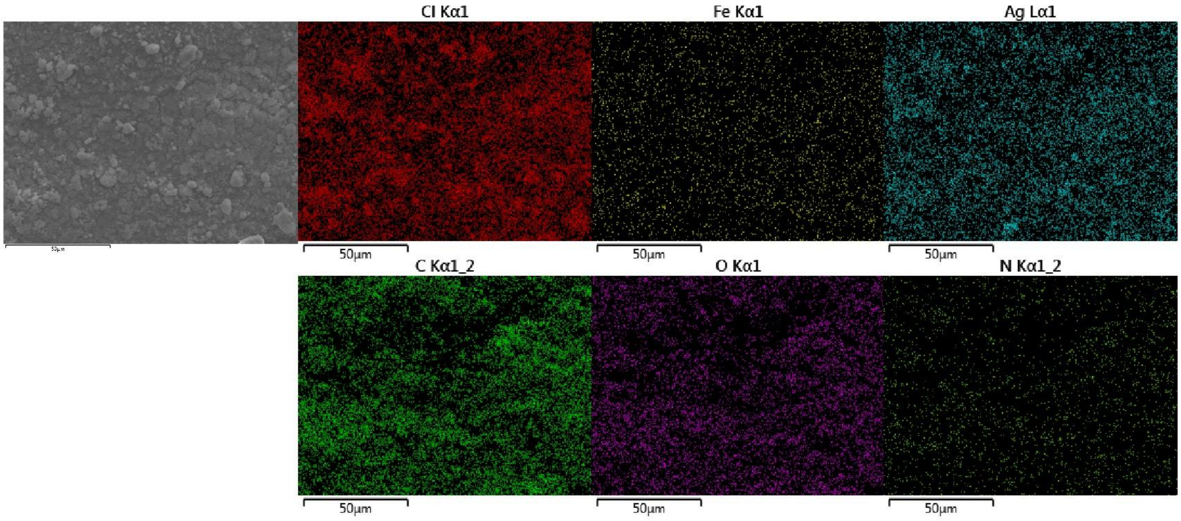

Supplementary Figure 10 SEM image and elemental mapping photographs of 1Ag (powder sample). The elemental mapping images show that Cl, Fe, Ag, C, O, and N are evenly distributed in the powder sample, indicating the presence of solvent chloroform molecules.

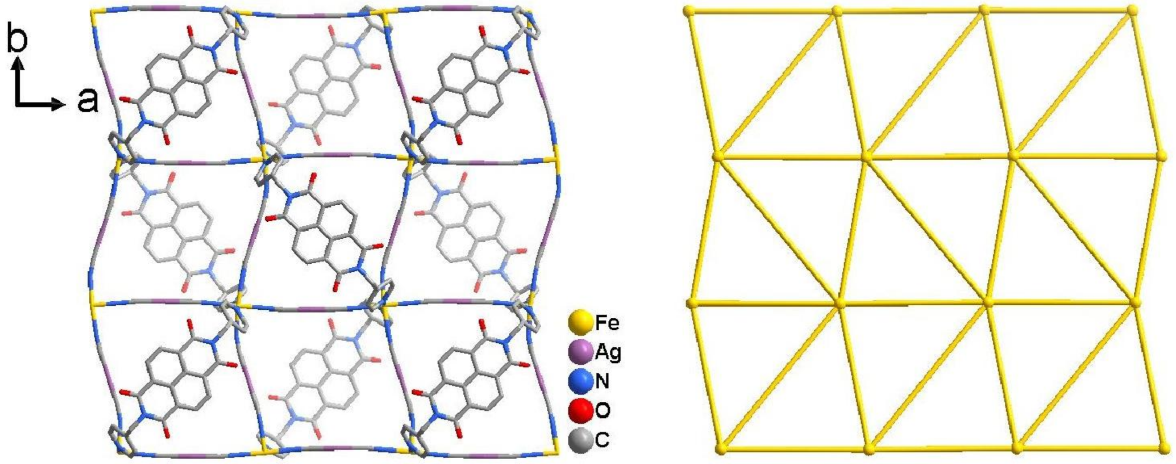

Supplementary Figure 11 2D layer with hxl topology for 1Ag. The 2D layered structure of **1Ag** (left) and its view for 6-connected **hxl** topology network (right).

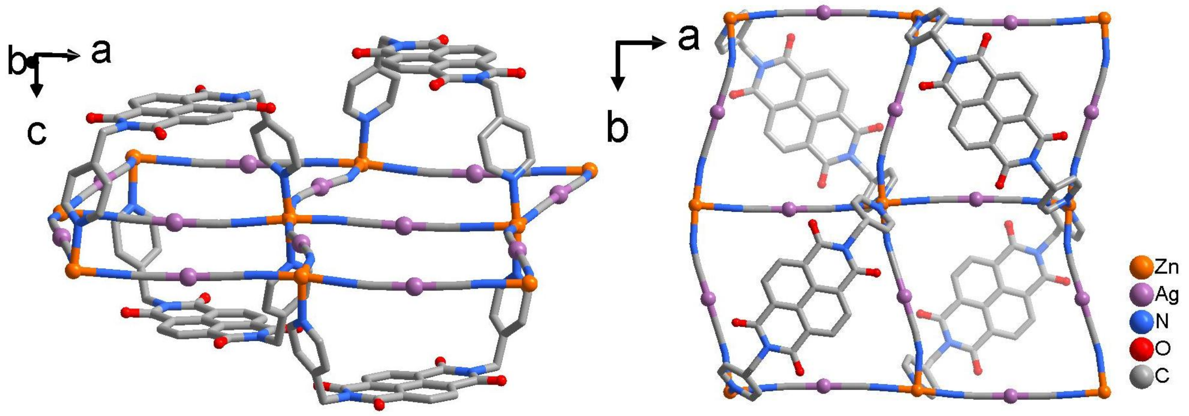

Supplementary Figure 12 The 2D layered structure of 2Ag. Left: side view. Right: top view. Hydrogen atoms and solvent molecules are omitted for clarity.

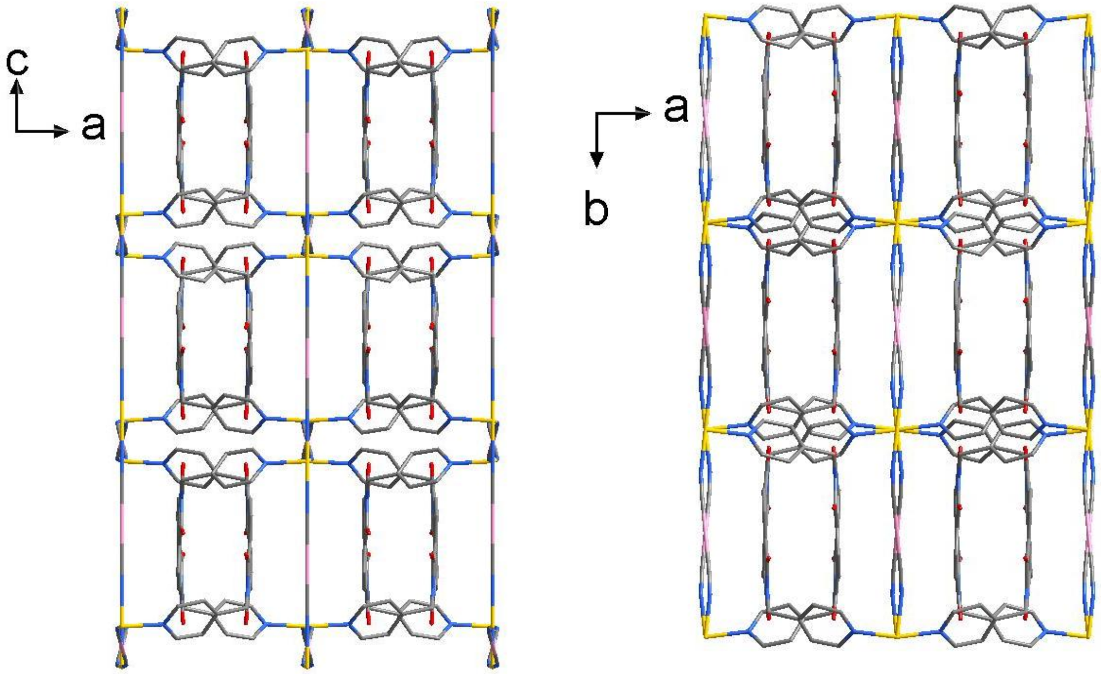

Supplementary Figure 13 Packing modes of 1Au. Cell packing diagram revealing interlayar π-π stacking along the *b*- and *c*-axis direction for **1Au**. Hydrogen atoms and solvent molecules are omitted for clarity.

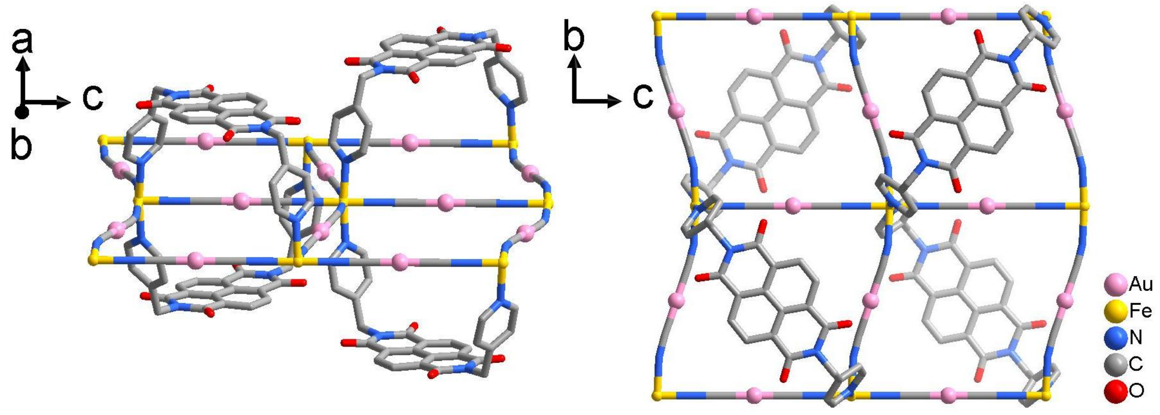

Supplementary Figure 14 The 2D layered structure of 1Au. Left: side view. Right: top view. Hydrogen atoms and solvent molecules are omitted for clarity.

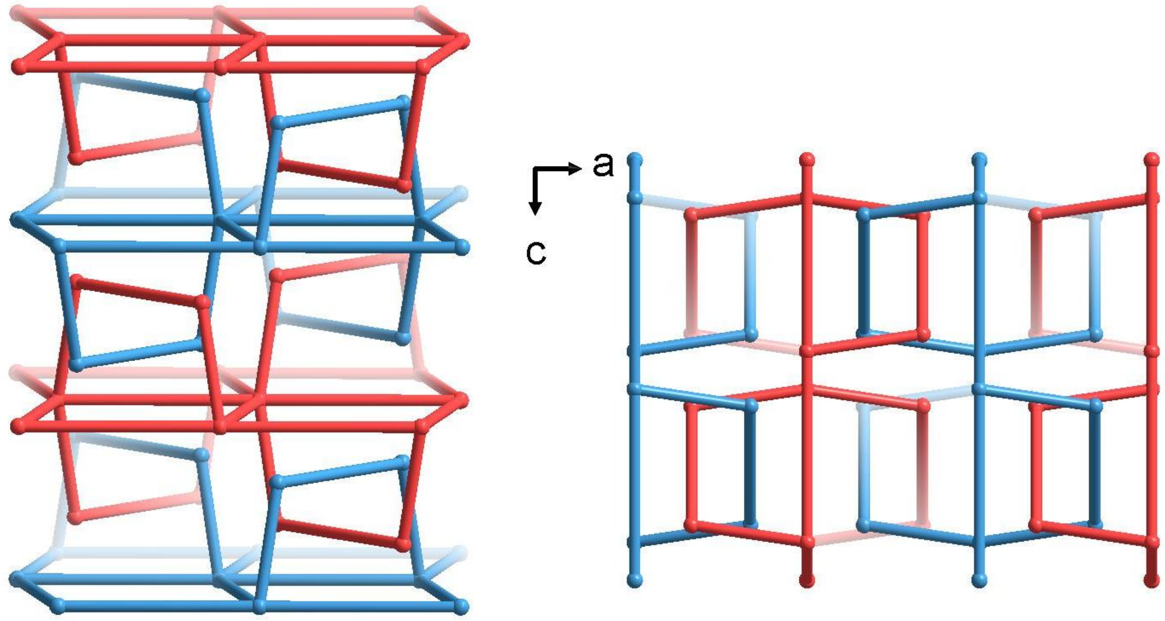

Supplementary Figure 15 Packing mode and entanglement of 2D layers for 1Au. Adjacent interlocking 2D layers are distinguished by red and blue.

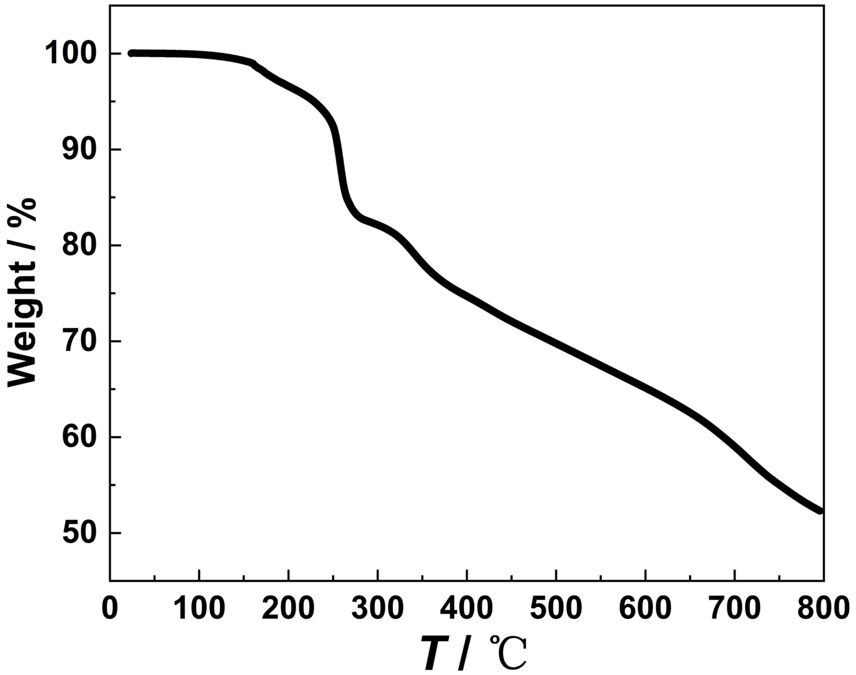

Supplementary Figure 16 Thermogravimetric analysis of 1Au. The desolvation process at high temperature (> 270 ºC) is accompanied by the weight loss due to decomposition.

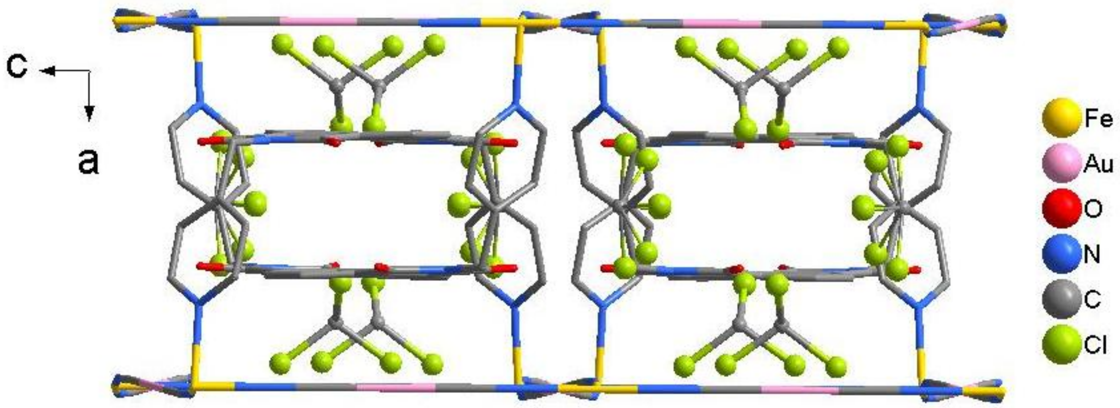

Supplementary Figure 17 Packing modes of 1Au. Cell packing diagram reveals interlayer π-π stacking along the *b*-axis direction for **1Au**. Hydrogen atoms are omitted for clarity.

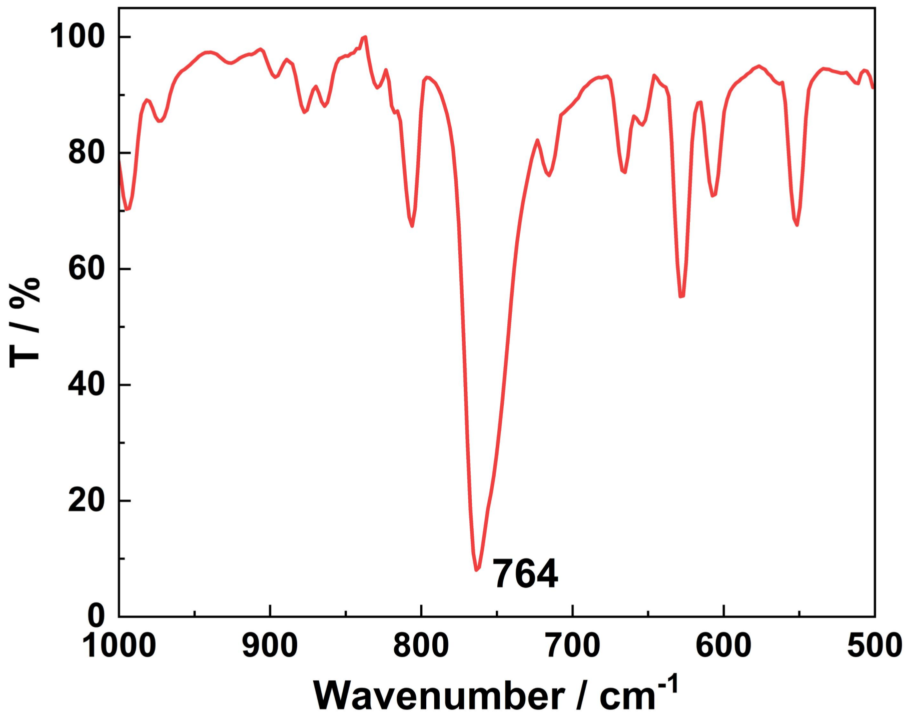

Supplementary Figure 18 IR spectrum of 1Au. The presence of chloroform is verified by the strong peak for C–Cl stretching vibration at 764 cm^{-1}.

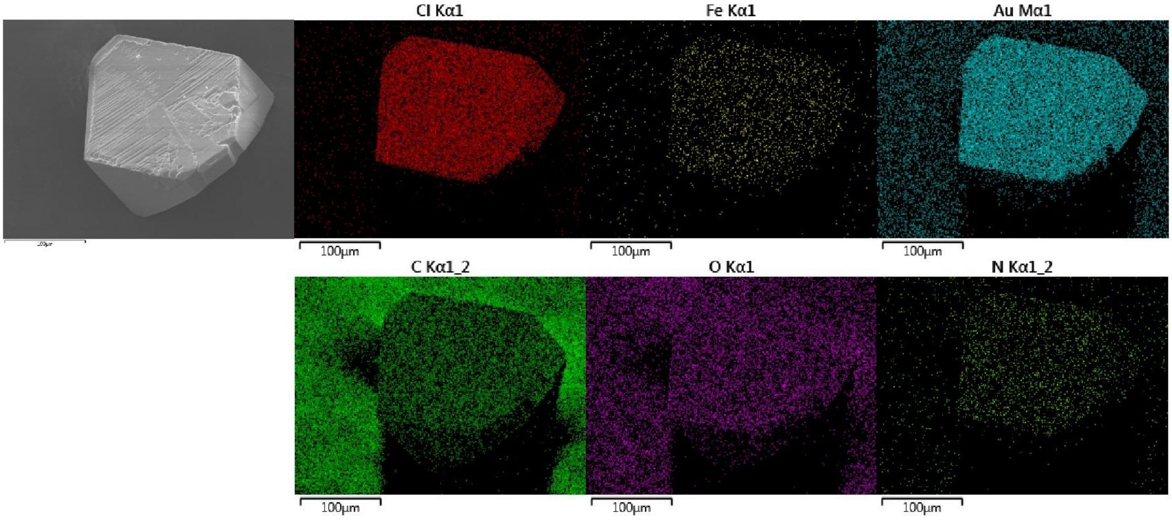

Supplementary Figure 19 SEM image and elemental mapping photographs of 1Au (single crystal). The elemental mapping images show that Cl, Fe, Au, C, O, and N are evenly distributed in the cystal, indicating the presence of solvent chloroform molecules.

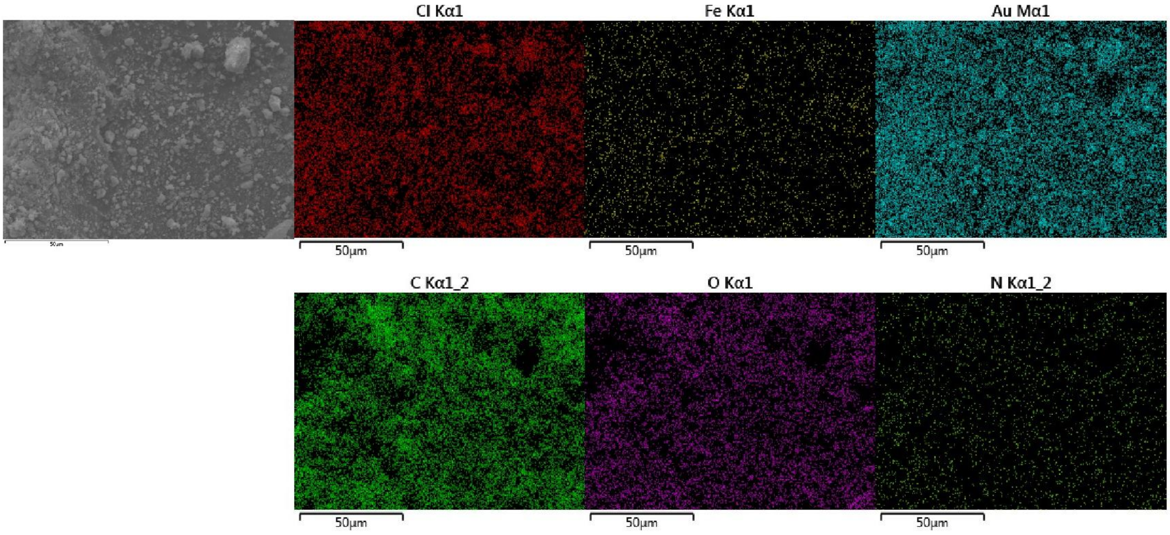

Supplementary Figure 20 SEM image and elemental mapping photographs of 1Au (powder sample). The elemental mapping images show that Cl, Fe, Au, C, O, and N are evenly distributed in the powder sample, indicating the presence of solvent chloroform molecules.

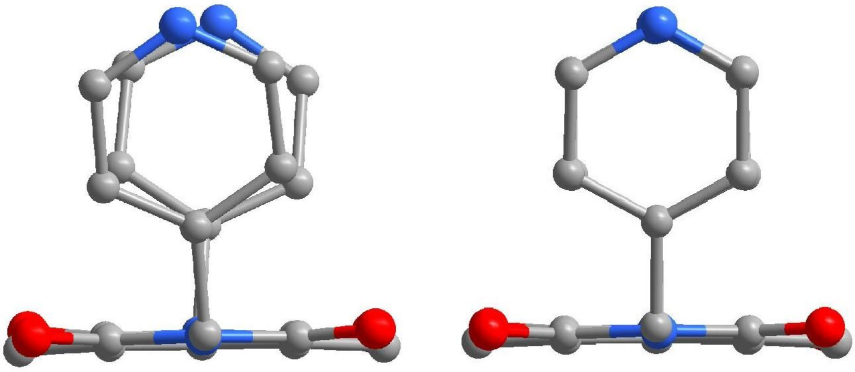

Supplementary Figure 21 Two BPND ligands in 1Ag. The asymmetric unit in **1Ag** contains two BPND ligands with different rotational angles.

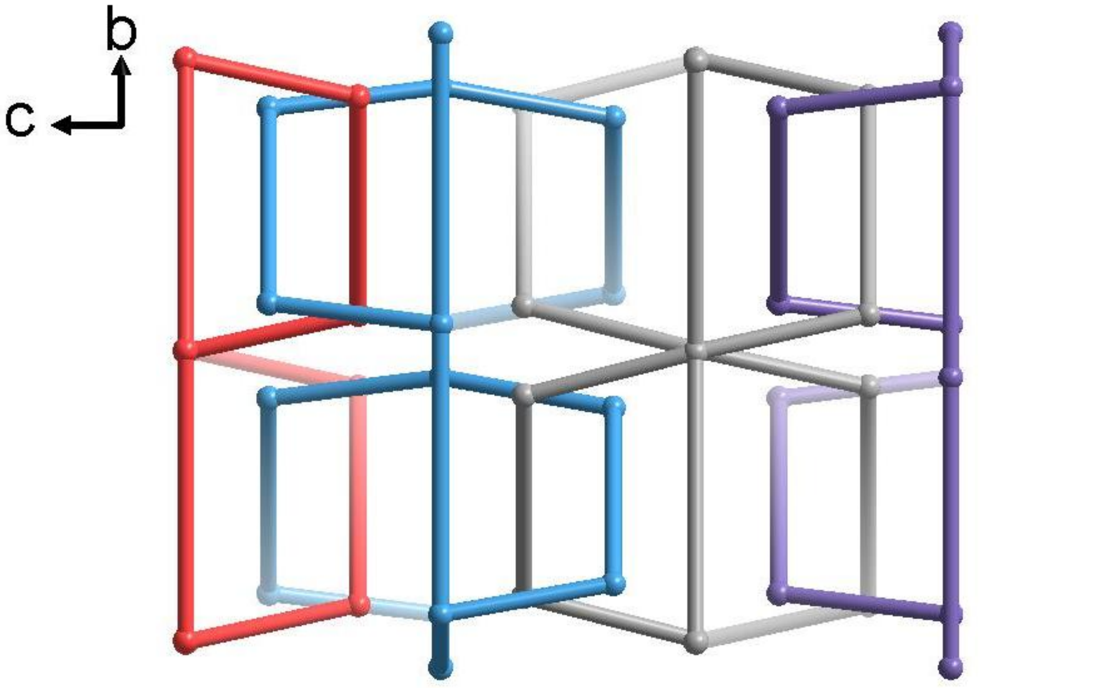

Supplementary Figure 22 Packing mode and entanglement of 2D layers for 1Ag. Four colors (red, blue, gray and purple) are used to illustrate adjacent interlocking 2D layers.

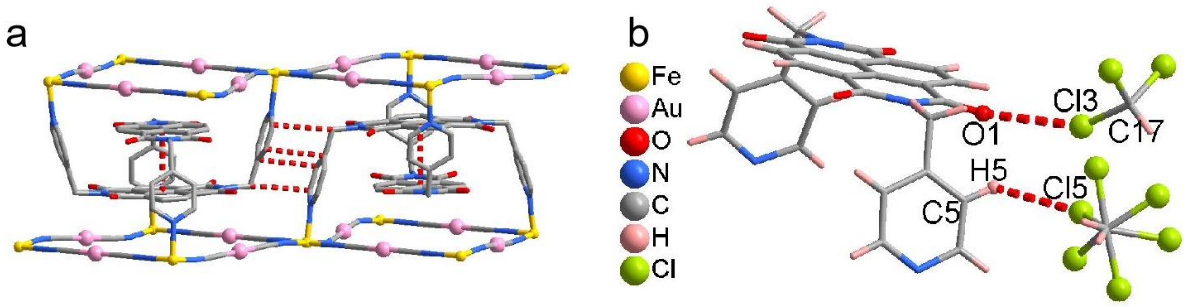

Supplementary Figure 23 Supramolecular interactions in 1Au. Supramolecular π-π stacking (a), C-Cl···O and C–H···Cl interactions (b) in **1Au** at 100 K. Red dashed lines represent supramolecular π-π stacking, C-Cl···O and C–H···Cl interactions.

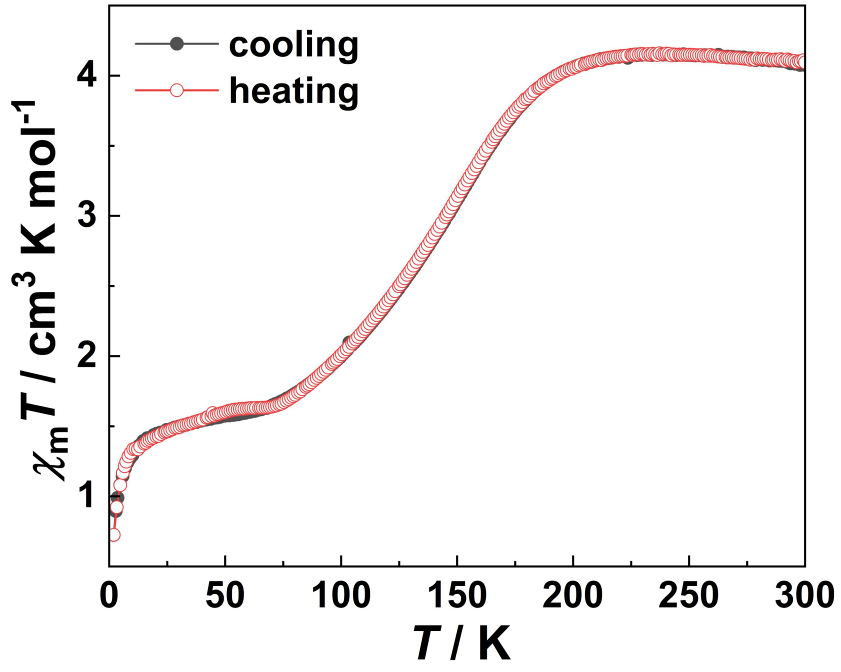

Supplementary Figure 24 Magnetic properties of 1Ag. Temperature-dependent magnetic susceptibilities for **1Ag** with a scan rate of 2 K min^{-1}.

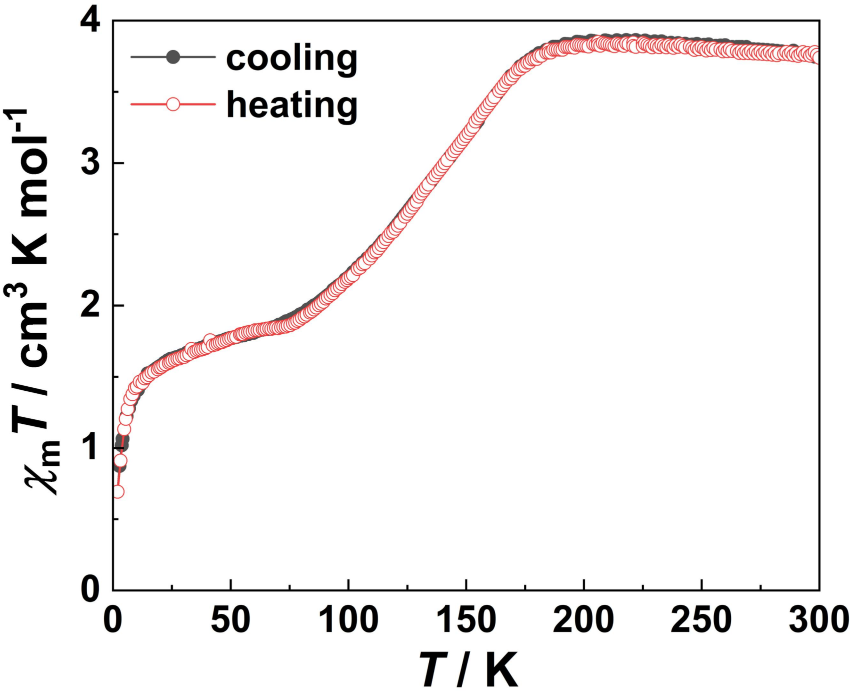

Supplementary Figure 25 Magnetic properties of 1Au. Temperature-dependent magnetic susceptibilities for **1Au** with a scan rate of 2 K min^{-1}.

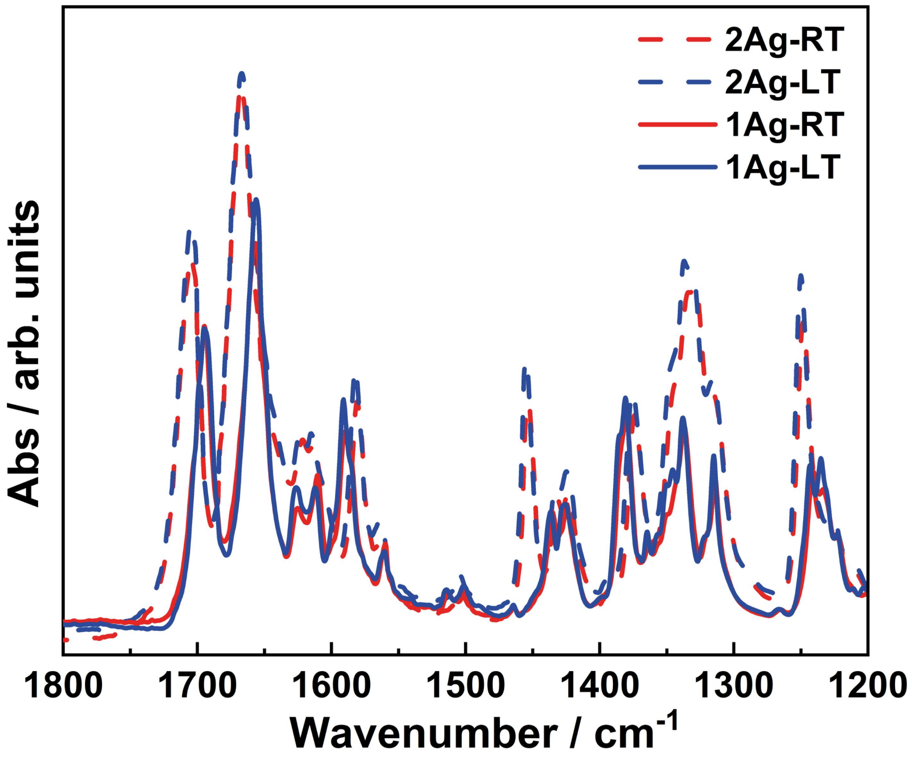

Supplementary Figure 26 Comparison of IR absorption spectra for 1Ag and 2Ag. IR absorption spectra for **1Ag** and **2Ag** in the range of 1200 - 1800 cm^{-1} at room temperature and 77 K.

Supplementary Table 6 Cartesian Coordinates of the truncated molecular model of 1Ag in the HS state for *ab initio* calculations.

Atom number	Element	X	Y	Z	Atom number	Element	X	Y	Z
1	Fe	4.311	5.2242	27.6109	23	C	3.7974	4.0311	32.4148
2	N	6.4118	5.0246	27.6157	24	C	4.7236	3.3869	31.6377
3	N	2.225	5.4793	27.752	25	H	5.1994	2.6502	31.9765
4	N	4.1721	3.0501	27.3358	26	H	5.6014	3.4038	29.8182
5	N	4.6036	7.4075	27.671	27	H	2.5744	4.2163	25.1775
6	N	4.2963	4.8626	29.7965	28	C	2.6986	5.2267	23.4318
7	N	4.09	5.4835	25.3948	29	H	1.964	4.812	23.0192
8	Ag	9.574	4.9406	27.8308	30	C	3.4354	6.1824	22.7541
9	C	7.5401	4.9783	27.7189	31	C	4.5215	6.7465	23.406
10	C	11.6238	4.9783	27.8257	32	H	5.0562	7.3822	22.9677
11	Ag	4.987	-0.0585	27.4113	33	H	5.5677	6.7507	25.1333
12	C	4.3826	1.9366	27.3248	34	C	1.0988	5.5467	27.8257
13	C	5.5867	-2.0482	27.5568	35	C	4.9383	8.4768	27.5568
14	N	12.75	5.0457	27.752	36	Ag	-0.951	5.5844	27.8308
15	N	5.9214	-3.1175	27.671	37	Ag	5.538	10.4665	27.4113
16	C	3.429	5.5088	30.5732	38	C	-2.9849	5.5467	27.7189
17	C	4.951	3.8416	30.3375	39	C	6.1424	12.4616	27.3248
18	C	3.0607	4.8857	24.7356	40	N	-4.1132	5.5004	27.6157
19	C	4.8205	6.3676	24.7135	41	N	6.3529	13.5752	27.3358
20	H	2.9786	6.254	30.2234	42	H	3.206831	6.440871	21.879715
21	C	3.1575	5.1257	31.8881	43	H	3.605216	3.731858	33.284926
22	H	2.5428	5.6119	32.4074					

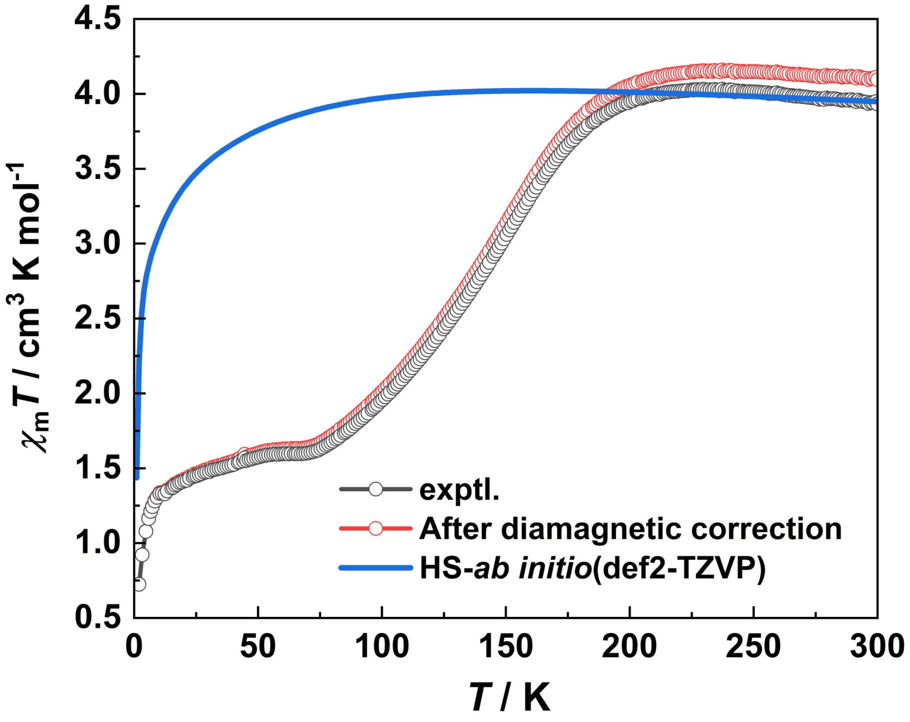

Supplementary Figure 27 Comparison of experimental and theoretical $\chi_m T$ values for 1Ag. The experimental $\chi_m T$ curves of **1Ag** and $\chi_m T$ values (solid blue line) obtained from *ab initio* calculations.

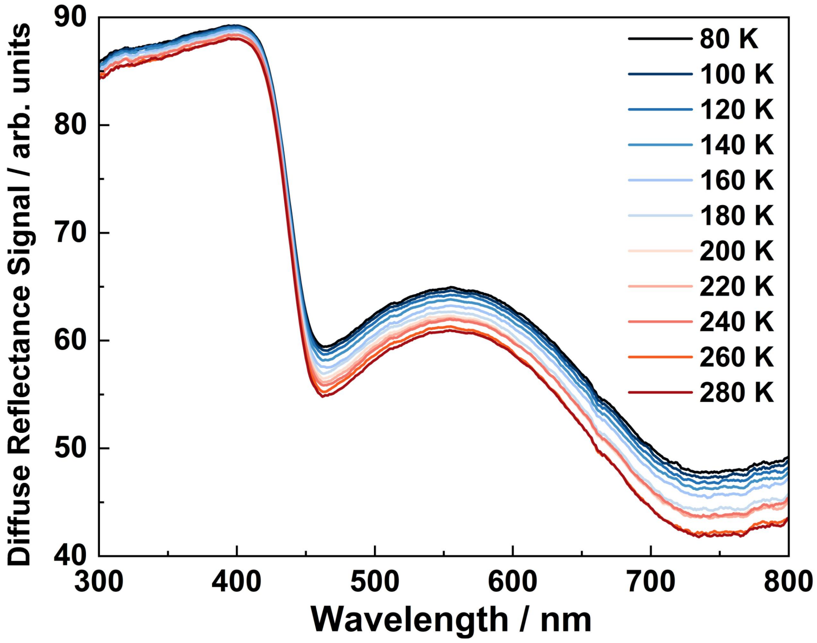

Supplementary Figure 28 Variable-temperature UV-vis diffuse reflectance spectroscopy of 1Ag. The UV-vis spectra of **1Ag** at different temperatures (in the heating mode) indicate that the absorption band centered at 555 nm in the visible region is temperature dependent.

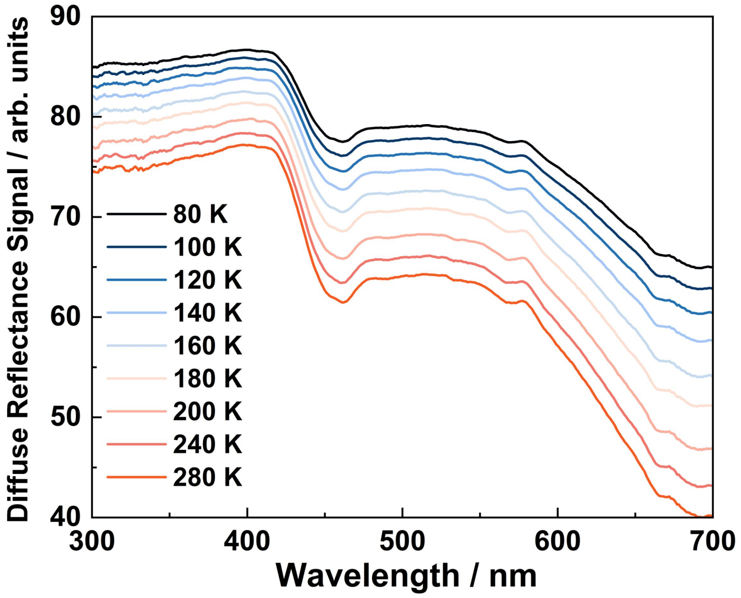

Supplementary Figure 29 Variable-temperature UV-vis diffuse reflectance spectroscopy of 1Au. The UV-vis spectra of **1Au** at different temperatures (in the heating mode) indicate that the absorption band at 525 nm in the visible region is temperature dependent.

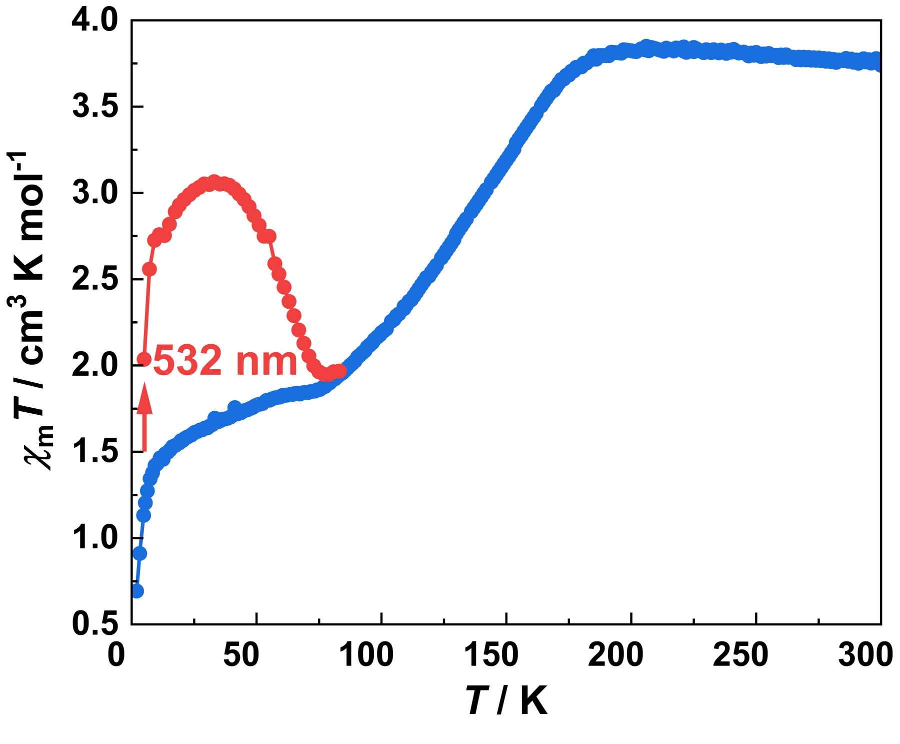

Supplementary Figure 30 Light-induced spin transition of 1Au. Temperature-dependent magnetic susceptibilities before (blue) and after irradiation (red) with 532 nm laser light in the heating mode for **1Au**.

Supplementary Table 7 Mössbauer spectroscopic parameters for 1Ag.

T	Isomer shift (δ / mm s^{-1})	Quadrupole splitting (ΔE_Q / mm s^{-1})	Area (%)	Assignment
RT	1.061	0.565	100	HS
50 K	1.147	0.999	31.72	HS
	0.499	0.457	68.28	LS

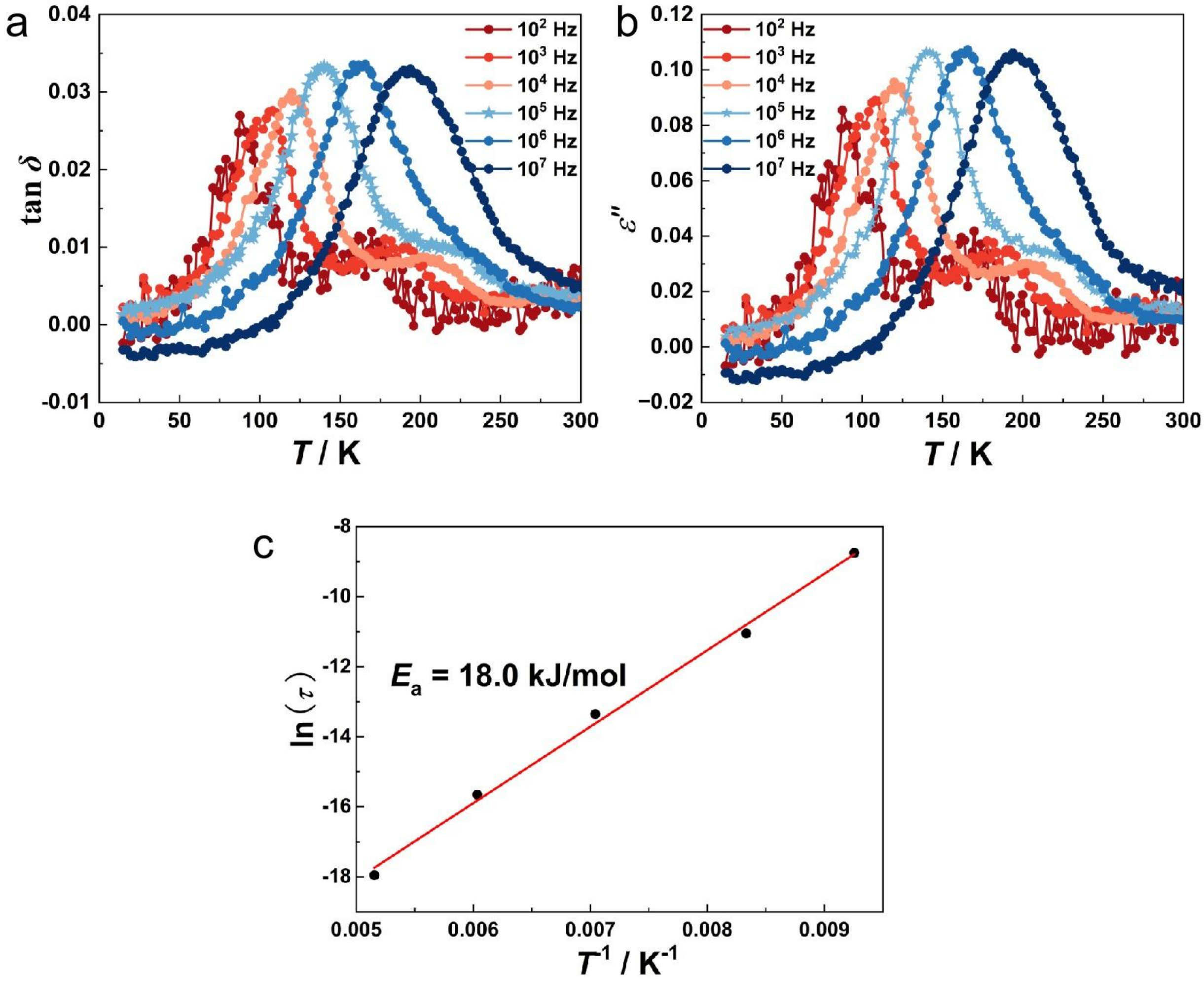

Supplementary Figure 31 Temperature-dependent dielectric properties of 1Ag. Temperature-dependent dielectric loss (tan δ) (a), the imaginary part of the dielectric constant (ε'') (b) under different frequencies (f = 100 Hz to 10 MHz) for **1Ag** measured in the heating mode at a scan rate of 2 K min^{-1}, and Arrhenius fitting of ln(τ) vs. T^{-1} plots (c).

Supplementary Table 8 Selected bond angles (°) for 1Ag at different temperatures.

T / K	100	170	250
N1-Fe1-N3	88.0(3)	88.4(3)	88.3(2)
N1-Fe1-N4#1	88.1(4)	87.5(3)	87.7(2)
N1-Fe1-N5	89.4(4)	89.0(3)	89.3(3)
N1-Fe1-N6	95.4(4)	96.0(3)	96.4(3)
N2#2-Fe1-N3	93.4(3)	93.8(3)	93.8(3)
N2#2-Fe1-N4#1	90.6(4)	90.6(3)	90.6(3)
N2#2-Fe1-N5	88.0(4)	87.4(3)	87.0(3)
N2#2-Fe1-N6	87.2(4)	87.8(3)	87.4(3)
N3-Fe1-N6	90.5(3)	89.6(3)	89.1(3)
N4#1-Fe1-N6	86.2(4)	86.1(3)	85.7(2)
N5-Fe1-N3	87.4(3)	87.5(3)	87.8(3)
N5-Fe1-N4#1	96.3(4)	97.2(3)	97.8(3)

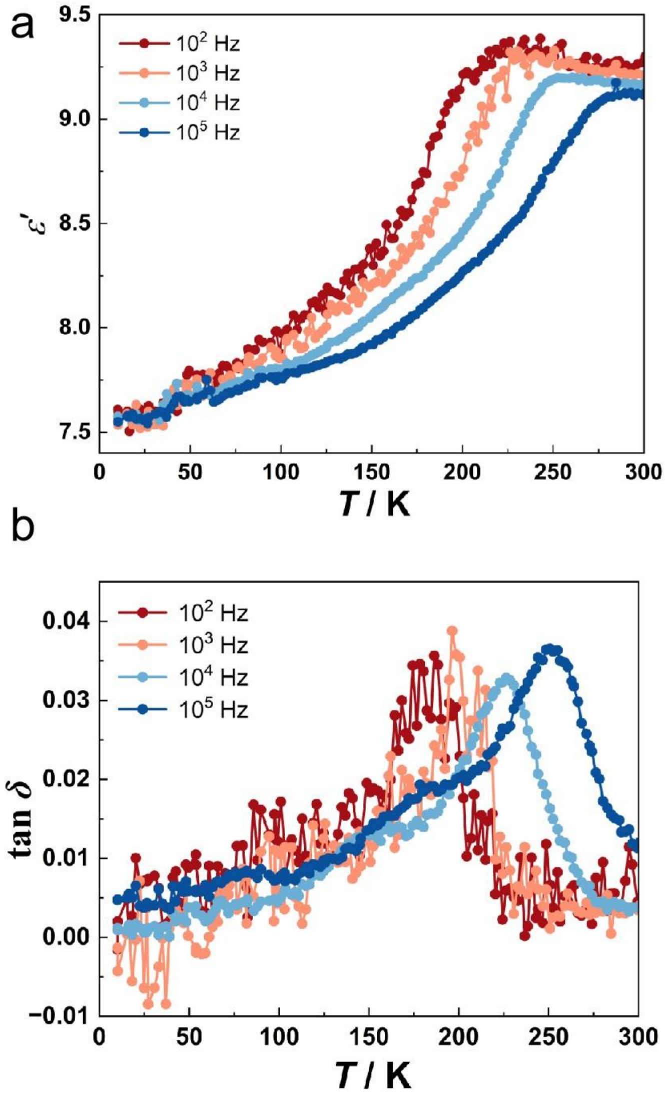

Supplementary Figure 32 Temperature-dependent dielectric properties of 1Au. Temperature-dependent dielectric constant (ε') (a) and dielectric loss (tan δ) (b) versus frequency plots (f = 100 Hz to 10^5 Hz) of **1Au** measured in the heating mode at a scan rate of 2 K min^{-1}.

Supplementary Table 9 Selected angles (°) for 1Au at different temperatures.

100 K		220 K	
N1-Fe1-N4#1	91.5(3)	N1-Fe1-N3#1	87.8(2)
N1#1-Fe1-N4#1	88.8(3)	N1-Fe1-N3	87.8(2)
N1#1-Fe1-N4	91.5(3)	N1-Fe1-N4	92.74(15)
N1-Fe1-N4	88.8(3)	N1-Fe1-N4#1	92.74(15)
N2-Fe1-N1	92.4(3)	N2#2-Fe1-N3	92.2(2)
N2-Fe1-N1#1	92.4(3)	N2#2-Fe1-N3#1	92.2(2)
N2-Fe1-N4#1	86.9(3)	N2#2-Fe1-N4#1	87.26(15)
N2-Fe1-N4	86.9(3)	N2#2-Fe1-N4	87.26(15)
N3#2-Fe1-N1#1	87.6(3)	N4-Fe1-N3#1	91.2(2)
N3#2-Fe1-N1	87.6(3)	N4-Fe1-N3	89.0(2)
N3#2-Fe1-N4	93.1(2)	N4#1-Fe1-N3#1	89.0(2)
N3#2-Fe1-N4#1	93.1(2)	N4#1-Fe1-N3	91.2(2)

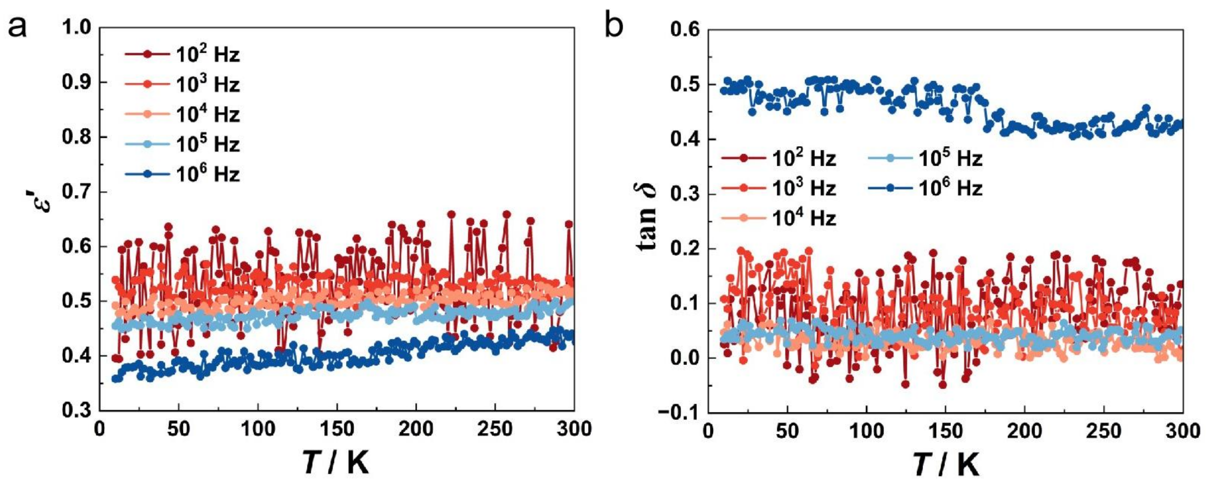

Supplementary Figure 33 Temperature-dependent dielectric properties of 2Ag. Temperature-dependent dielectric constant (ε') (a) and dielectric loss (tan δ) (b) versus frequency plots (f = 100 Hz to 1 MHz) of **2Ag** measured in the heating mode at a scan rate of 2 K min^{-1}.

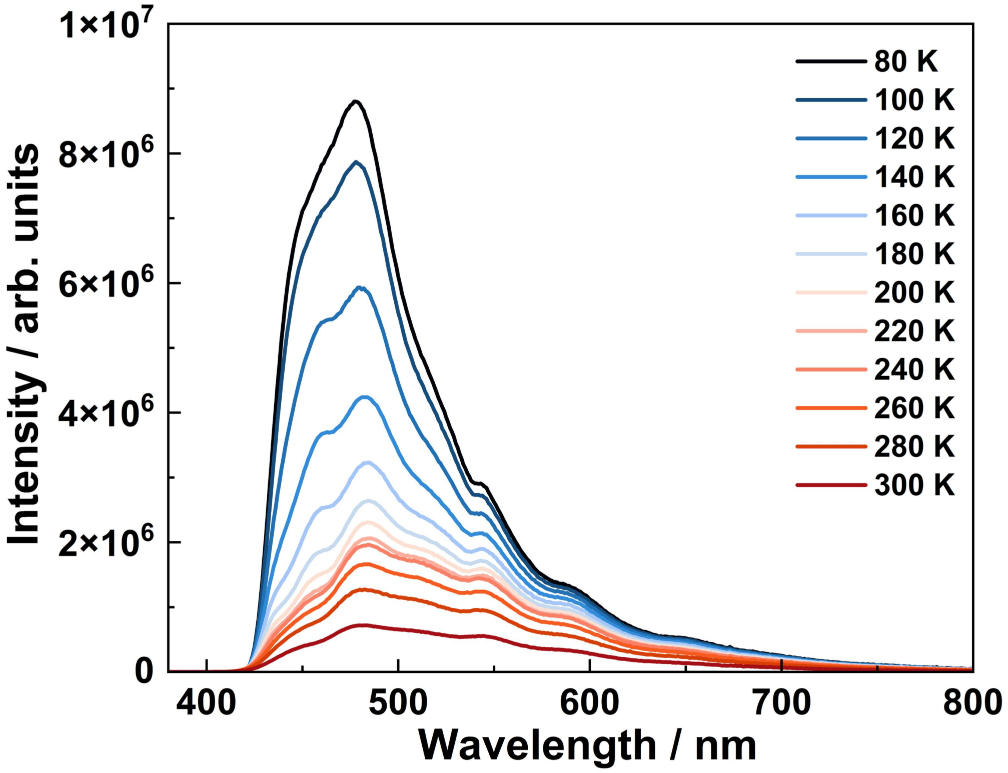

Supplementary Figure 34 Photoluminescent properties of the BPND ligand. Temperature-dependent emission spectra (λ_{ex} = 355 nm) in the heating mode for the BPND ligand.

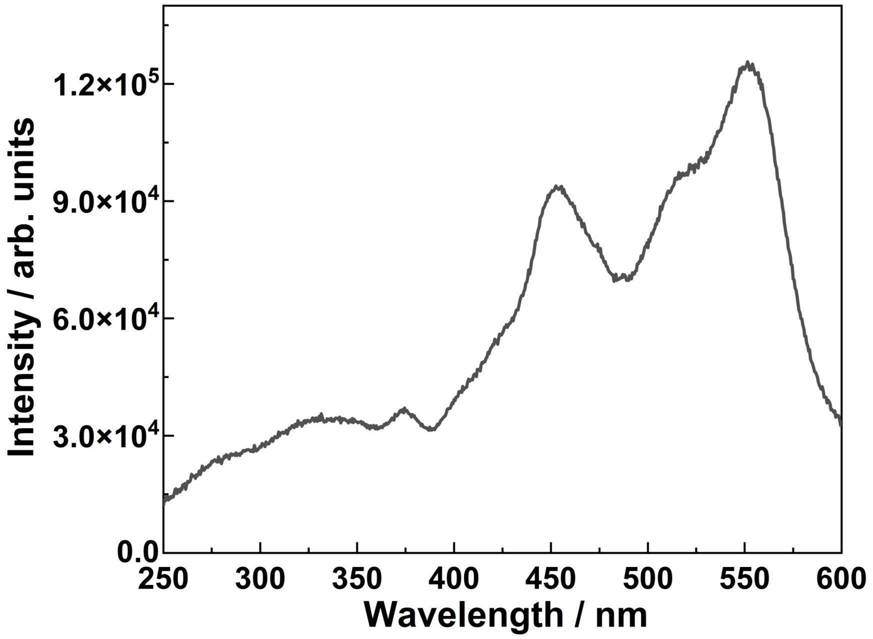

Supplementary Figure 35 Excitation spectrum for 1Ag. The room-temperature excitation spectrum for **1Ag**.

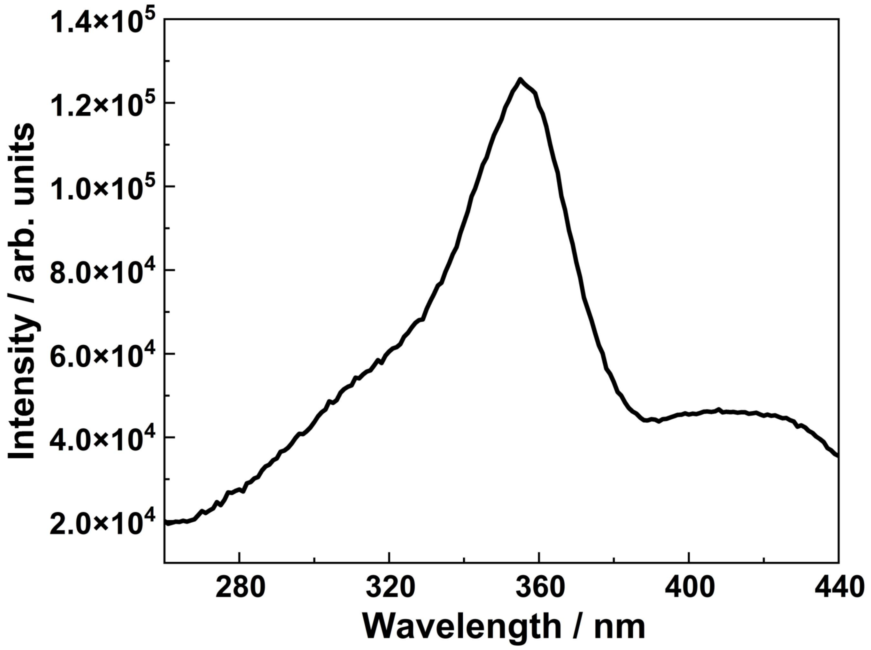

Supplementary Figure 36 Excitation spectrum for 1Au. The room-temperature excitation spectrum for **1Au** with a maximum excitation wavelength of 355 nm.

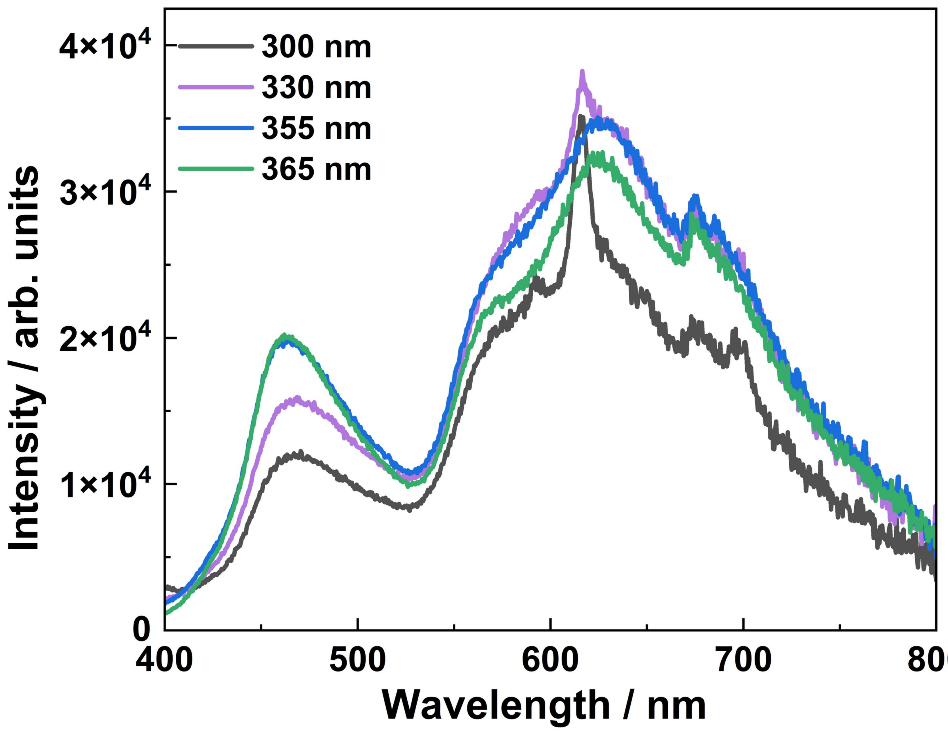

Supplementary Figure 37 Emission spectrum for 1Ag. The room-temperature emission spectra under different excitation lights (300 nm, 330 nm, 355nm and 365 nm) for **1Ag**.

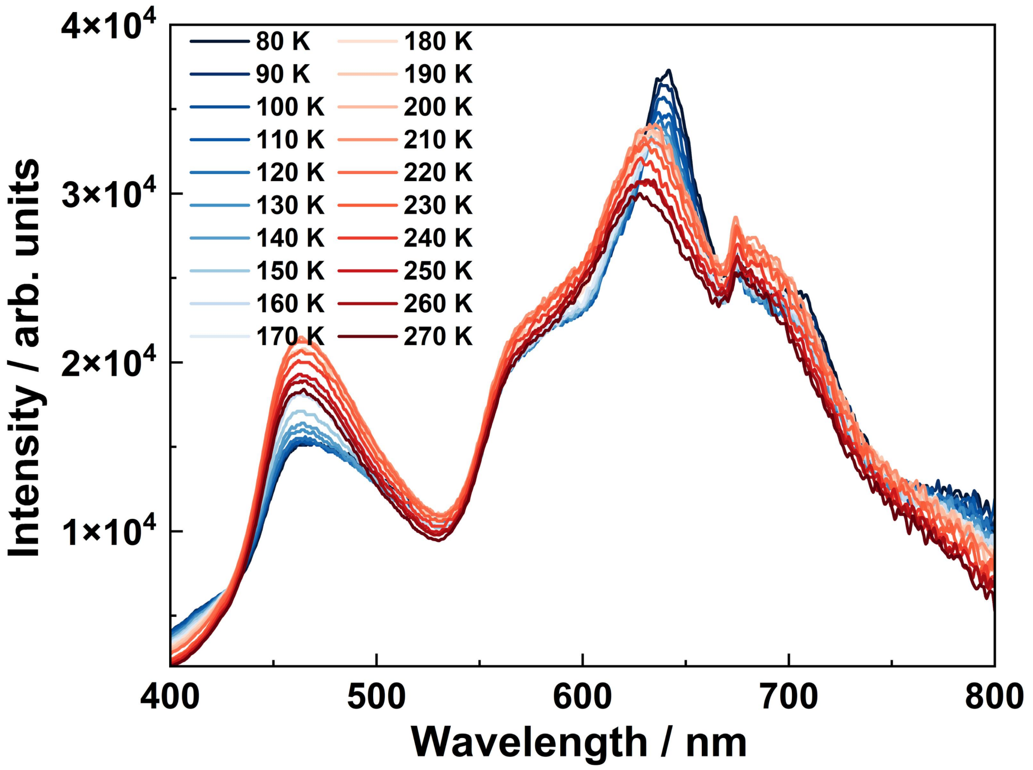

Supplementary Figure 38 Photoluminescent properties of 1Ag. Temperature-dependent emission spectra (λ_{ex} = 355 nm) in the heating mode for **1Ag**.

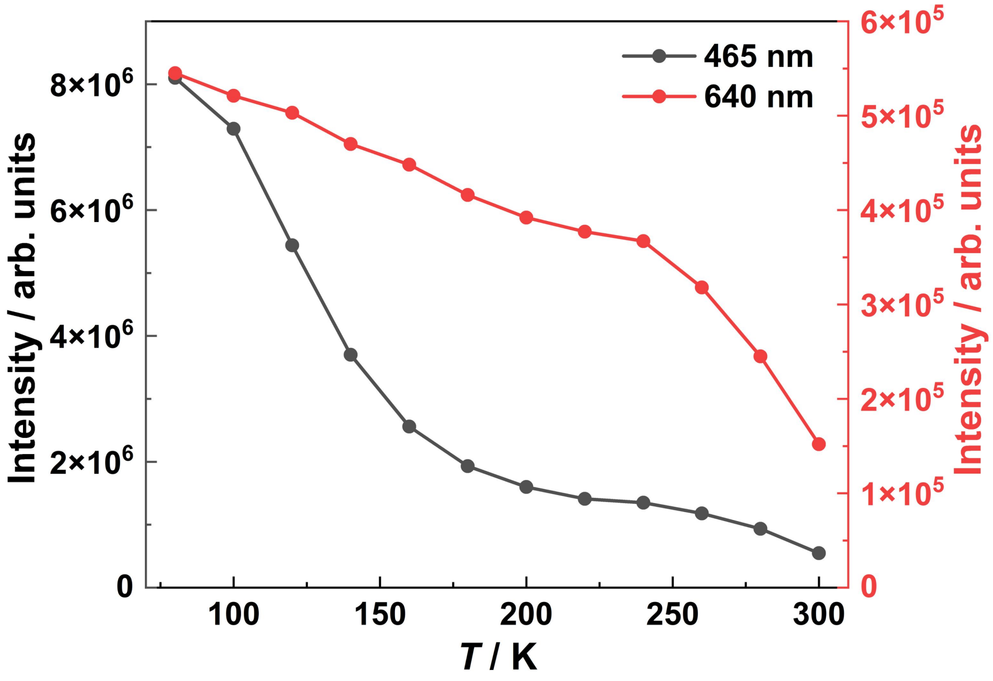

Supplementary Figure 39 Temperature-dependent emission intensity for BPND. Temperature dependence of the emission intensity at 465 nm and 640 nm for the free ligand BPND.

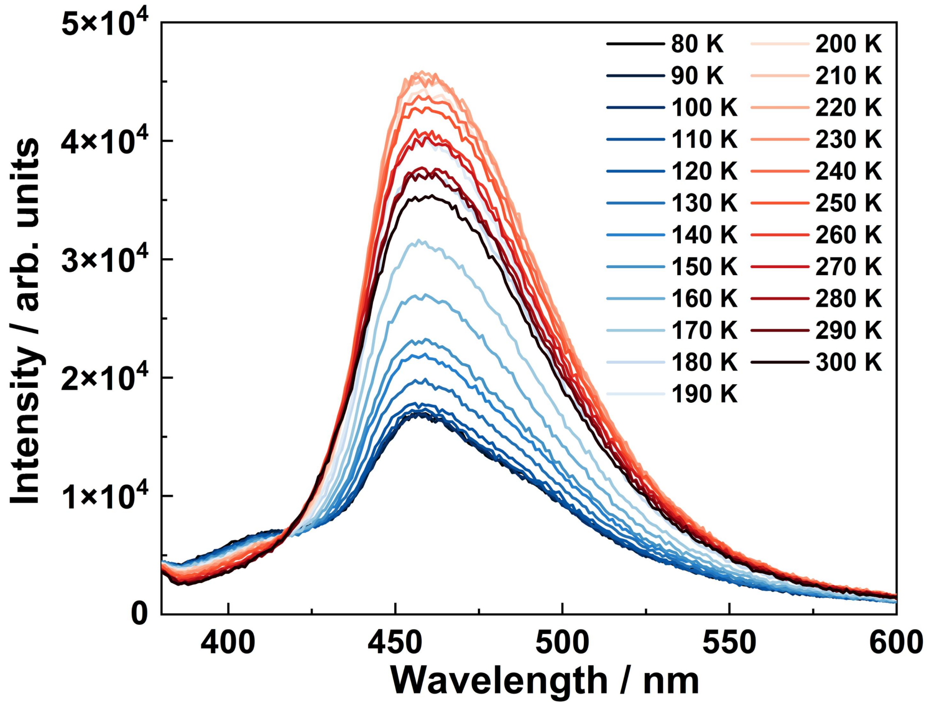

Supplementary Figure 40 Photoluminescent properties of 1Au. Temperature-dependent emission spectra (λ_{ex} = 355 nm) in the heating mode for **1Au**.

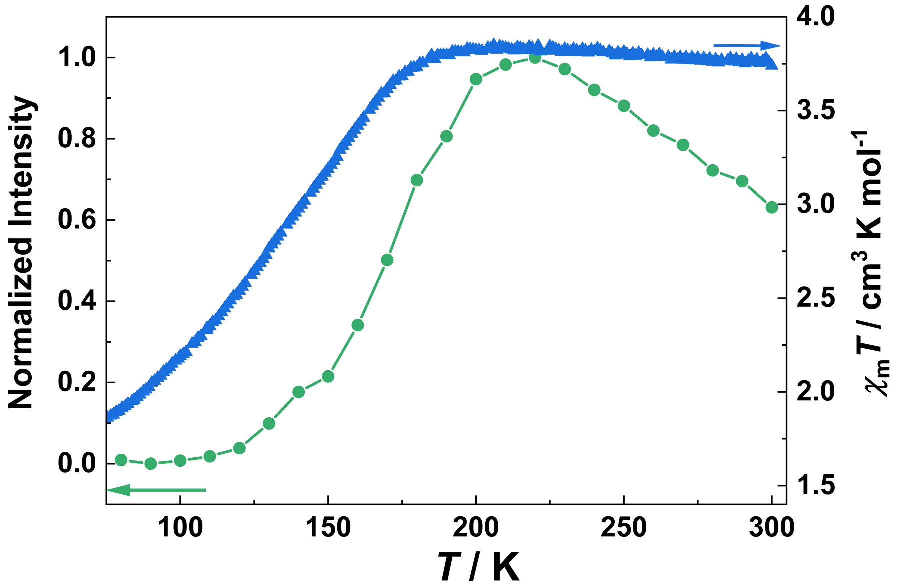

Supplementary Figure 41 Luminescence-SCO coupling effect of 1Au. $\chi_m T$ and normalized maximum emission intensity in the heating mode for **1Au**.

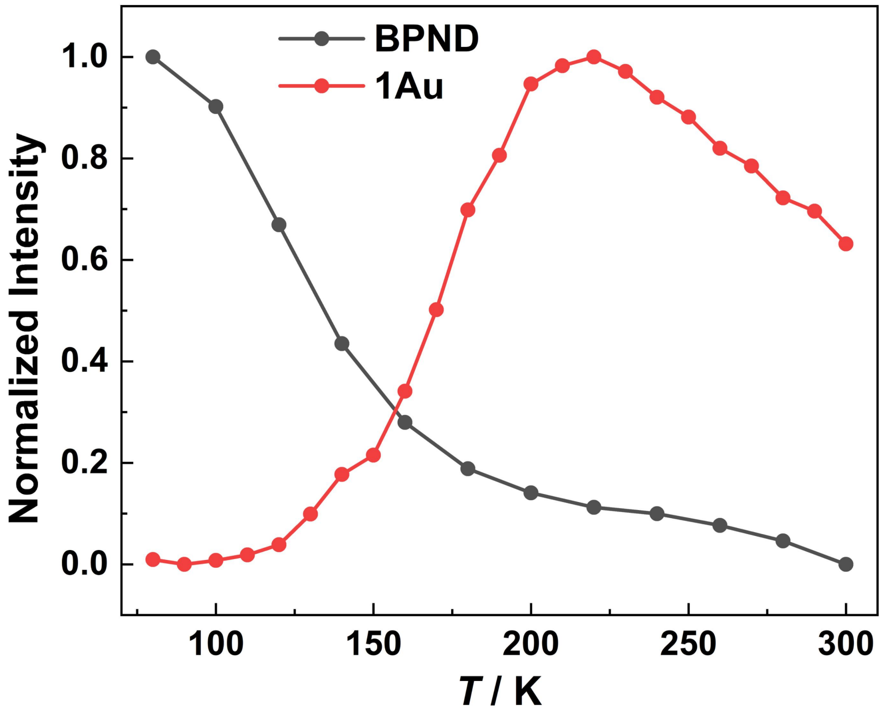

Supplementary Figure 42 Comparison of the emission intensity for 1Au and BPND. Corresponding changes in the emission intensity of BPND ligand and **1Au** at 460 nm with temperature.

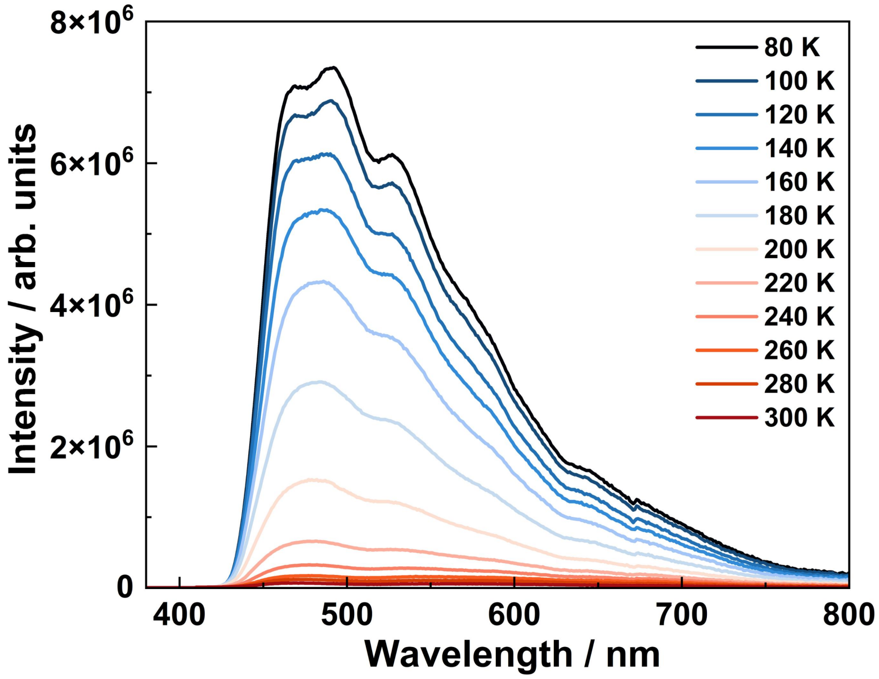

Supplementary Figure 43 Photoluminescent properties of 2Ag. Temperature-dependent emission spectra (λ_{ex} = 355 nm) in the heating mode for **2Ag** show monotonic decrease in the emission intensity.

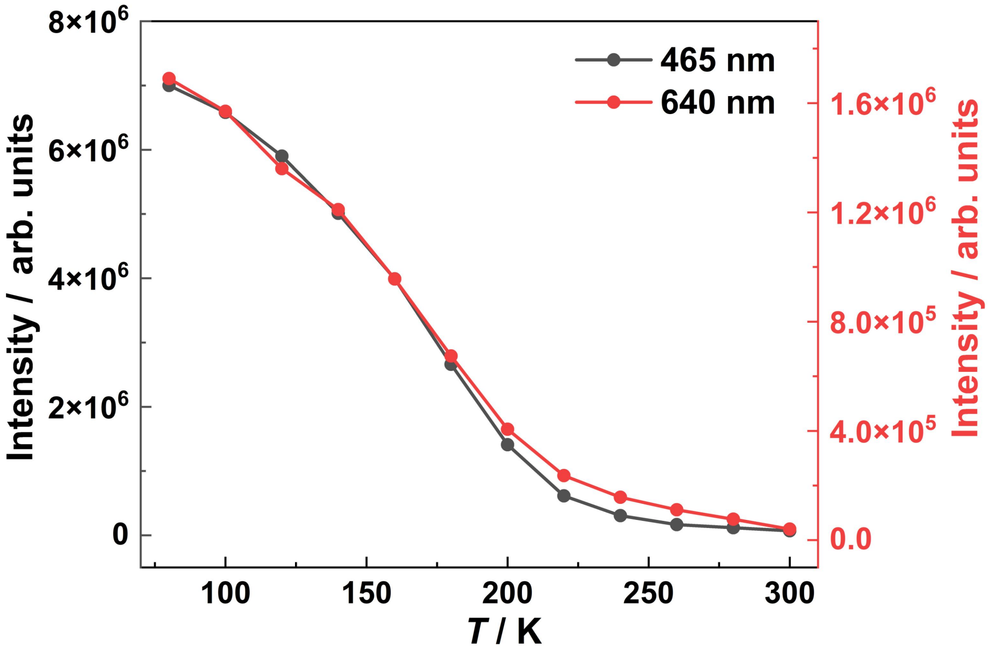

Supplementary Figure 44 Temperature-dependent emission intensity for 2Ag. The emission intensity of **2Ag** at 465 nm and 640 nm monotonically decreases with increasing temperature.

Supplementary Table 10 Selected excitation energies and corresponding oscillator strengths (*f*) / description for LS 1Ag and HS 1Ag.

Excited states (LS)	Oscillation strength (*f*) / Description	Excited states (HS)	Oscillation strength (*f*) / Description
2.976 eV / 416.7 nm	0.0291 / LL'CT	2.914 eV / 425.5 nm	0.0067 / LMCT
2.681 eV / 462.4 nm	0.1541 / MLCT	2.829 eV / 438.3 nm	0.0034 / MLCT
2.573 eV / 481.8 nm	0.0848 / MLCT	2.676 eV / 463.4 nm	0.0075 / LMCT
2.337 eV / 530.4 nm	0.0830 / MLCT	2.557 eV / 484.9 nm	0.0074 / LMCT
2.253 eV / 550.4 nm	0.2085 / MLCT	2.320 eV / 525.4 nm	0.0100 / MLCT, *d-d*
2.206 eV / 562.1 nm	0.0711 / MLCT	2.258 eV / 549.1 nm	0.0094 / LL'CT, $\pi \to \pi^*$

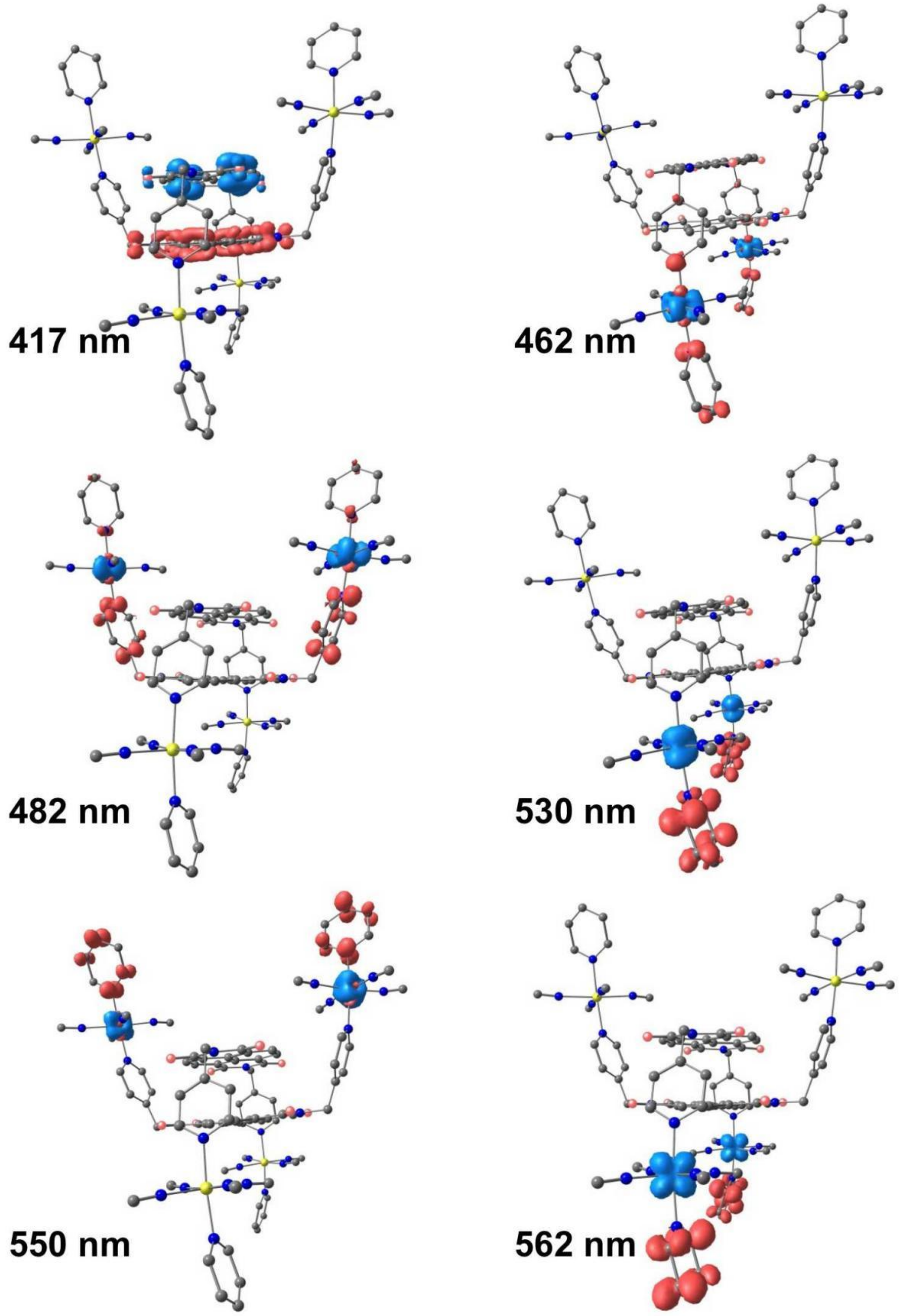

Supplementary Figure 45 Charge density difference of LS 1Ag. Charge density difference (red: increase in the electron density; blue: decrease in the electron density) between excited and ground states of LS **1Ag**. Value of contour envelopes is 0.0025 au.

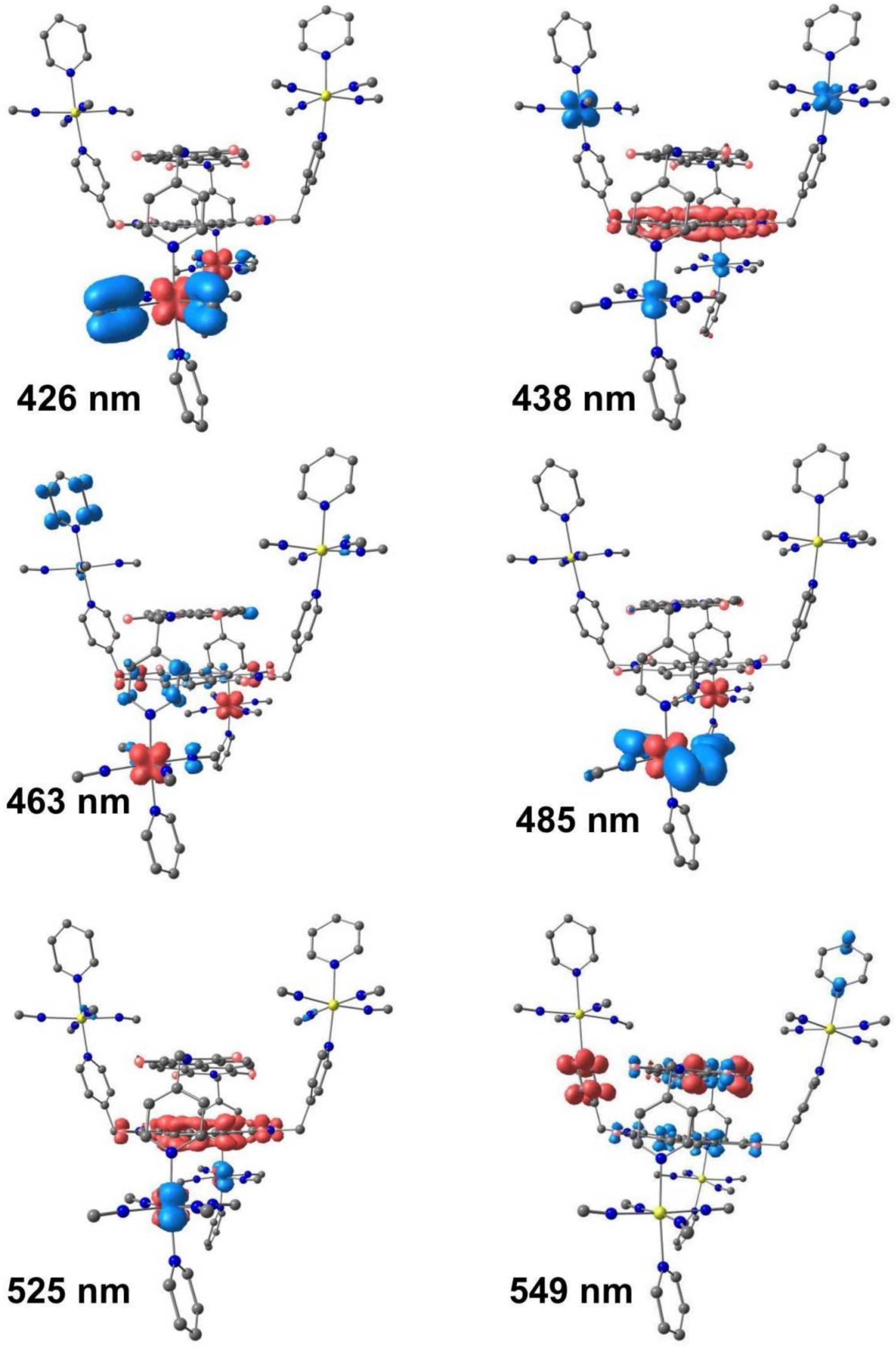

Supplementary Figure 46 Charge density difference of HS 1Ag. Charge density difference (red: increase in the electron density; blue: decrease in the electron density) between excited and ground states of HS **1Ag**. Value of contour envelopes is 0.0025 au.
