# Supplementary material for: Integrating spin-dependent emission and dielectric switching in FeII catenated metal-organic frameworks
Source: Nat Commun. 2024 May 10;15:3961. doi: 10.1038/s41467-024-48425-8 (PMC11087595; doi:10.1038/s41467-024-48425-8)
Supplement: Supplementary file 3 — Description of Additional Supplementary Files [file 41467_2024_48425_MOESM3_ESM.pdf]

Description of Additional Supplementary Files

File Name: Supplementary Data 1

Description: Cartesian coordinates of the truncated molecular model of 1Ag
